# Variant APOL1 protein in plasma associates with larger particles in humans and mouse models of kidney injury

**DOI:** 10.1371/journal.pone.0276649

**Published:** 2022-10-24

**Authors:** Michael Andrews, Teruhiko Yoshida, Clark M. Henderson, Hannah Pflaum, Ayako McGregor, Joshua A. Lieberman, Ian H. de Boer, Tomas Vaisar, Jonathan Himmelfarb, Bryan Kestenbaum, Joon-Yong Chung, Stephen M. Hewitt, Briana A. Santo, Brandon Ginley, Pinaki Sarder, Avi Z. Rosenberg, Taichi Murakami, Jeffrey B. Kopp, Zsuzsanna Kuklenyik, Andrew N. Hoofnagle

**Affiliations:** 1 Centers for Disease Control and Prevention, Atlanta, Georgia, United States of America; 2 Kidney Disease Section, Kidney Diseases Branch, National Institute of Diabetes and Digestive and Kidney Diseases, National Institutes of Health, Bethesda, Maryland, United States of America; 3 Department of Laboratory Medicine and Pathology, University of Washington, Seattle, Washington, United States of America; 4 Department of Medicine, University of Washington, Seattle, Washington, United States of America; 5 Kidney Research Institute, University of Washington, Seattle, Washington, United States of America; 6 Center for Cancer Research, NCI, NIH, Bethesda, Maryland, United States of America; 7 Department of Pathology and Anatomical Sciences, Jacobs School of Medicine & Biomedical Sciences, University at Buffalo, Buffalo, New York, United States of America; 8 Department of Pathology, Johns Hopkins Medical Institutions, Baltimore, Maryland, United States of America; 9 Department of Nephrology, Ehime Prefectural Central Hospital, Ehime, Japan; University of Illinois at Chicago, UNITED STATES

## Abstract

**Background:**

Genetic variants in apolipoprotein L1 (APOL1), a protein that protects humans from infection with African trypanosomes, explain a substantial proportion of the excess risk of chronic kidney disease affecting individuals with sub-Saharan ancestry. The mechanisms by which risk variants damage kidney cells remain incompletely understood. In preclinical models, APOL1 expressed in podocytes can lead to significant kidney injury. In humans, studies in kidney transplant suggest that the effects of APOL1 variants are predominantly driven by donor genotype. Less attention has been paid to a possible role for circulating APOL1 in kidney injury.

**Methods:**

Using liquid chromatography-tandem mass spectrometry, the concentrations of APOL1 were measured in plasma and urine from participants in the Seattle Kidney Study. Asymmetric flow field-flow fractionation was used to evaluate the size of APOL1-containing lipoprotein particles in plasma. Transgenic mice that express wild-type or risk variant APOL1 from an albumin promoter were treated to cause kidney injury and evaluated for renal disease and pathology.

**Results:**

In human participants, urine concentrations of APOL1 were correlated with plasma concentrations and reduced kidney function. Risk variant APOL1 was enriched in larger particles. In mice, circulating risk variant APOL1-G1 promoted kidney damage and reduced podocyte density without renal expression of APOL1.

**Conclusions:**

These results suggest that plasma APOL1 is dynamic and contributes to the progression of kidney disease in humans, which may have implications for treatment of APOL1-associated kidney disease and for kidney transplantation.

## Introduction

In humans, the gene encoding apolipoprotein L1 (*APOL1*) has two common polymorphic alleles, G1 (rs73885319, rs60910145) and G2 (rs71785313) [1, 2], which are found only in persons of sub-Saharan African ancestry. Carriers of one G1 or G2 polymorphism in *APOL1* are protected from African trypanosomiasis, also known as African sleeping sickness [3, 4]. However, persons who are homozygous or compound heterozygous for these alleles have a substantially greater risk of progressive kidney disease, particularly diseases caused by HIV or other precipitants of collapsing focal segmental glomerulosclerosis [1, 2, 5]. Most research directed at mechanisms by which APOL1 might cause kidney function decline has focused on endogenous expression within the kidneys, especially in podocytes [6–11]. Less attention has been paid to circulating plasma APOL1 protein, which may be due to several studies that associate kidney allograft failure to donor genotype [12–14] and to a study showing that plasma levels of risk-variant APOL1 did not associate with renal disease [15]. However, more recent data from a transplant study suggests that recipient genotype, and therefore circulating APOL1, may still be relevant [16].

The non-polymorphic (G0) variant of *APOL1* encodes a 44 kDa protein with the sequence LNILNNNYK at residues 382–390, which are near the C-terminus [17]. The G1 polymorphism is a missense mutation that leads to the substitution of isoleucine at position 384 with a methionine (LNMLNNNYK); a second substitution at position S342 (to glycine) is also present [18]. The G2 polymorphism is a short deletion of residues 388–389 (LNILNNK) [17]. It has been hypothesized that these sequence variations alter the conformation stability around the leucine zipper motif at the C-terminus and enhance the formation of pores in the cell membrane that lead to cytotoxicity [10, 11, 19–22], although other mechanisms have been proposed, including endoplasmic reticulum stress [23, 24], inflammasome activation [25], pyroptosis [6], protein kinase R activation [26], and mitochondrial dysfunction [27]. Other studies have demonstrated that risk variant APOL1 localizes to different membranes and lipid droplets when expressed in cell models compared to APOL1-G0, suggesting different intracellular biochemical characteristics and behavior [9, 28]. We previously reported that the amount the APOL1 residing on HDL particles was lower in participants with impaired kidney function and hypothesized that APOL1 resides on different particles at different stages of kidney function [29]. This change in extracellular biochemical behavior and the fact that the primary reservoir of APOL1 in the body is circulating in plasma made us revisit the hypothesis that risk variant APOL1 in plasma could help promote kidney injury and/or disease progression [29–31].

To test the hypotheses that risk variant APOL1 is biochemically different in plasma and contributes to the development and progression of proteinuric kidney diseases like focal segmental glomerulosclerosis and arterionephrosclerosis, we developed assays to quantify APOL1 in plasma, to measure the size of APOL1-containing particles in plasma, and to quantify APOL1 in urine. We also developed a novel mouse model that expresses *APOL1*-G0, *APOL1*-G1, or *APOL1*-G2 in the liver from an albumin promoter and used it to show that circulating APOL1 exacerbates damage to the kidney, particularly the G1 variant. Overall, our studies suggest plasma APOL1, even in the absence of podocyte derived APOL1, could contribute to kidney pathology.

## Materials and methods

A detailed description of the methods and materials used for these studies is included in **S1 File**. Brief descriptions are provided here.

### Human samples

Plasma, urine, and DNA samples were obtained from participants in the Seattle Kidney Study (**SKS**), an IRB-approved cohort study described previously [32]. Race was self-identified, estimated glomerular filtration rate (**eGFR**) was calculated from plasma creatinine using the 2021 CKD-EPI equation without a race coefficient included [33], and serum total protein and urine albumin-to-creatinine ratios were determined using an automated clinical chemistry analyzer (Beckman DxC 800, Beckman Coulter, Brea, CA). Amounts of APOL1 in the HDL fraction of plasma were determined previously [29]. In addition to samples from SKS participants, four fresh human plasma samples were acquired from Interstate Blood Bank (Memphis, TN) as quality control materials for size fractionation experiments.

### Quantification of total APOL1 and phenotyping of APOL1 in human plasma

APOL1 protein was quantified in plasma using trypsin digestion and LC-MS/MS [Waters Acquity I-Class system (Milford, MA) and Waters TQ-S micro, **Table A in S1 File**]. The assay was calibrated using external calibrators comprised of pooled human serum diluted into chicken serum [34]. The isoforms of APOL1 present in each plasma sample were also determined by LC-MS/MS by comparing the ratio of each polymorphic APOL1 peptide (LNILNNNYK or LNMLNNNYK) to a non-polymorphic APOL1 peptide (VTEPISAESGEQVER) with empirical minimum cutoffs to detect the presence of peptide vs. absence of peptide, and therefore each haplotype. From these cut-offs, the *APOL1* genotype was identified for each participant. While this is a relatively new approach to determining the genotype of the individual, it is similar to other published studies determining the genotype of *APOL1* and vitamin D binding protein [34, 35].

### APOL1 genotyping

The *APOL1* G0, G1 (rs73885319), and G2 (rs71785313) alleles were detected by multiplexed qPCR with TaqMan allele-specific primer/probe genotyping assays (ThermoFisher Scientific, Waltham, MA). We used a commercially available set of primers and probes that distinguish the G0 and G1 alleles (ThermoFisher Assay ID: C__98253221_10). To distinguish G0 from G2 alleles, a custom genotyping assay (Assay ID: AN7DPWD) was designed using ThermoFisher’s custom TaqMan SNP assay design tool following the manufacturer’s instructions. Genotyping in 98 participants was used to cross-validate the phenotyping approach, which demonstrated a 100% agreement between genotype and phenotype.

### Size fractionation, particle size determination, and protein analysis

Particles in plasma samples were separated using an asymmetric flow field-flow fractionation (**AF4**) system (AF2000, PostNova Analytics, Salt Lake City, UT) with a UV detector. Forty fractions were collected for each sample. For each fraction, the average hydrodynamic size of particles was determined by dynamic light scattering using a DynaPro plate reader (Wyatt Technologies, Santa Barbara, CA, **Fig A in S1 File**). Proteins in each fraction were then quantified with in-line trypsin digestion and LC-MS/MS (Shimadzu Scientific Instruments/Perfinity Biosciences, West Lafayette, IN). Peptides were monitored using a 6500 QTRAP (AB Sciex, Foster City, CA, USA) in multiple reaction monitoring mode (**Table A in S1 File**). The experimental concentration vs. hydrodynamic size profiles were deconvolved using JMP software (SAS Institute, Cary, North Carolina).

### Quantification of APOL1 in human urine

Proteins in urine were precipitated with ice-cold acetone/hydrochloric acid. APOL1 was quantified in dried precipitates using trypsin digestion and LC-MS/MS. Heavy-labeled APOL1 internal standard peptide was added prior to digestion.

### Transgenic mice and the triple intervention model

We generated Alb/APOL1 transgenic mice on the FVB/N background. These mice carry the human *APOL1* gene under the control of the murine albumin promoter. We generated three strains: Alb/APOL1-G0, Alb/APOL1-G1 and Alb/APOL1 G2, each of which contains the coding sequence for one of these common *APOL1* genetic variants. All mouse experiments were conducted in accordance with the National Institutes of Health Guide for the Care and Use of Laboratory Animals and were approved in advance by the NIDDK Animal Care and Use Committee (Animal study proposal, K097-KDB-17 & K096-KDB-20). We euthanized mice by cervical dislocation after anesthesia with intraperitoneal 10% tribromethanol (Avertin) injection and after confirming loss of sensation by toe pinching. One Alb/APOL1-G1 mouse died on day 10 of the experimental period and was excluded from analysis (the body weight of this mouse was not different from the other mice). We induced podocyte injury and proteinuria using a triple intervention approach, including administration of interferon-γ, puromycin aminonucleoside, and basic fibroblast growth factor [26]. In this study, we used interferon-γ, in addition to puromycin aminonucleoside, and basic fibroblast growth factor, to be consistent with our previous work [26] and due to its potential contribution to podocyte injury [36]. We collected urine at day -2, day 7, and day 14 for 24 h using metabolic cages. On day 14, mice were euthanized and plasma, serum, kidneys, and liver were collected for analyte measurement and histologic analysis.

### Mouse urinary biomarkers and glomerular filtration rate (GFR)

We determined urine albumin levels with Albuwell M ELISA kits (Ethos Biosciences, Newtown Square, PA). Urine creatinine concentrations were measured using the Creatinine Companion kit (Ethos Biosciences). Urine NGAL concentrations were measured using the Mouse Lipocalin-2/NGAL DuoSet ELISA (R&D Systems, Minneapolis, MN). We measured GFR ten days after puromycin aminonucleoside injection by monitoring the disappearance of administered FITC-sinistrin (Medi Beacon, St. Louis, MO) with a fluorescence sensor (NIC-Kidney, Mannheim Pharma & Diagnostics, Mannheim, Germany). Fluorescent signals were analyzed according to the manufacturer’s instructions [37].

### Analysis of mouse tissues

Chromogenic *in situ* detection of transgene RNA was performed on tissue microarrays prepared from formalin-fixed paraffin-embedded blocks, using RNAScope (Advanced Cell Diagnostics, Biotechne, Minneapolis, MN), the RNA probe-Hs-*APOL1*-O1 (Advanced Cell Diagnostics), and the RNAScope 2.5 HD Reagent Kit [38]. For immunohistochemistry, tissues were fixed in 10% buffered formalin for 24 h, embedded in paraffin, and sectioned at 4–5 μm. After sectioning, the tissues were deparaffinized/rehydrated and antigens were retrieved with citrate-buffered medium in a hot water bath. APOL1 protein was detected with the primary antibody (5.17D12, rabbit monoclonal), which was kindly provided by Genentech (South San Francisco, CA), [11] the ImmPRESS HRP Universal Antibody (Horse Anti-Mouse/Rabbit IgG) Polymer Detection Kit, and the ImmPACT DAB EqV Peroxidase Substrate (Vector Laboratories, Burlingame, CA) protocol. Slides were counter-stained with hematoxylin. For histological analysis, tissues were stained with periodic acid-Schiff stain and evaluated by a nephropathologist (AZR). The percentage of sclerosed glomeruli (both segmentally and globally sclerosed) was determined for each mouse. A total of 72–367 glomeruli were evaluated per mouse. The number of injured tubules per area of renal cortex was also determined for each mouse. Podocyte density in individual glomeruli was determined by PodoCount, a computational tool for whole-slide podocyte quantification [39] after labeling for p57^kip2^, a marker of podocyte terminal differentiation (ab75974, Abcam, Cambridge, UK), detection with horse radish peroxidase (RU-HRP1000, Diagnostic BioSystems, Pleasanton, CA) and diaminobenzidine chromogen substrate (BSB0018A, Bio SB, Santa Barbara, CA), and post-staining with a periodic acid-Schiff stain without hematoxylin counterstain [40]. The method uses a combination of stain deconvolution [41], digital image processing [42, 43], and simple feature engineering [44, 45] to compute histologic morphometrics [46, 47] from glomeruli and resident podocyte nuclei in whole-slide images of kidney specimens.

### Statistical analysis

Normality was assessed using the Shapiro-Wilk test and parameters were log-transformed when needed. The following parameters were log-transformed: concentrations of APOL1 in plasma and urine, eGFR, plasma cholesterol, plasma triglycerides, plasma HDL cholesterol, urine albumin-to-creatinine ratio, age, and body mass index. Medians were compared with the Wilcoxon test. Means were compared with the Student’s t-test. The Shapiro-Wilk tests, Wilcoxon test, Student’s t-tests, and linear regressions were performed within the R statistical environment (v4.0.0).

### Study approval

SKS was approved by the Human Subjects Division at the University of Washington (#25422). All participants provided written informed consent. The analysis of the distributions of lipoprotein particles was performed at the Centers for Disease Control and was approved as research not involving identifiable human subjects. The mouse studies were performed at the National Institutes of Health in accordance with the National Institutes of Health Guide for the Care and Use of Laboratory Animals and were approved in advance by the NIDDK Animal Care and Use Committee (Animal study proposal, K097-KDB-17 & K096-KDB-20).

## Results

### Plasma concentrations of APOL1 are associated with race and eGFR, but not APOL1 genotype

**Fig 1:**
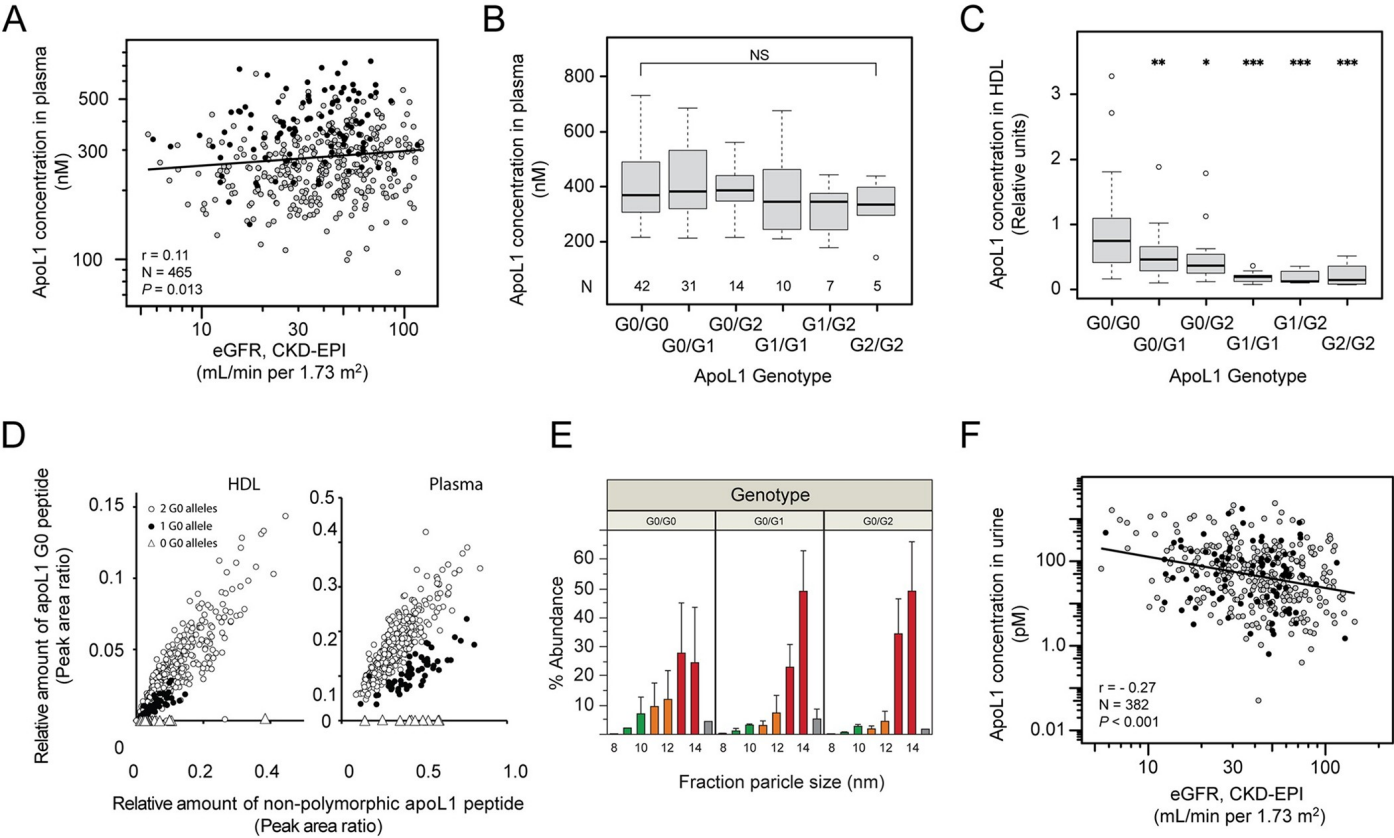
APOL1 in human plasma and urine. (A) The concentration of APOL1 in plasma (nM, determined by LC-MS/MS) is correlated with eGFR (mL/min/1.73 m^2^, calculated by the CKD-EPI 2021 equation). Black participants are represented with solid black circles and non-Black participants are represented with gray circles. A total of 465 participants had plasma for APOL1 analysis and eGFR from the same baseline study visit. The Pearson correlation coefficient for the entire cohort is shown. Statistical significance was determined by linear regression of the log-log transformed data. (B) The total concentration of APOL1 in plasma is shown for each *APOL1* genotype in self-described Black participants. The number of participants (N) with each genotype is listed. (C) The relative concentration of APOL1 in the HDL fraction of plasma is shown for each *APOL1* genotype in self-described Black participants. Statistical significance for each genotype vs. G0/G0 was assessed using Student’s t-test. *P<0.05, **P<0.01, ***P<0.001. (D) Risk variant APOL1 causes co-expressed APOL1-G0 to shift out of the HDL fraction of plasma. The relative amount of G0-specific peptide (*i*.*e*., the peptide that has a different amino acid sequence in each of APOL1-G0, APOL1-G1, and APOL1-G2) is plotted vs. a non-polymorphic APOL1 peptide from a different region of the protein for individuals who were heterozygous for risk variant *APOL1* alleles (closed circles), homozygous for *APOL1*-G0 (open circles), or homozygous for risk variant alleles (open triangles). (E) Lipoprotein particles in plasma were separated using asymmetric flow field-flow fractionation and analyzed using LC-MS/MS. The relative amount of APOL1 protein in each fraction (%) is shown as mean ± SD. Bars are shaded different colors in order to more conveniently compare size fractions across genotypes. The data for the G0/G0 genotype are derived from samples from an equal number of Black and non-Black participants. All heterozygous participants self-described as Black race. (F) The amount of APOL1 in urine is negatively correlated with eGFR. The concentration of APOL1 in urine was measured using LC-MS/MS. Black participants are represented with solid black circles and non-Black participants are represented with gray circles. There were 382 participants in SKS with urine available for APOL1 analysis who also had eGFR measured at the same baseline study visit. The Pearson correlation coefficient is shown. Statistical significance was determined by linear regression of the log-log transformed data.

Previous human studies have suggested that plasma concentrations of APOL1 are not associated with eGFR or chronic kidney disease [15, 30]. Since this contrasted with our previous observations that the amount of APOL1 in HDL is lower in participants with reduced eGFR, we developed an LC-MS/MS method to quantify the concentration of APOL1 in bulk plasma and determine the *APOL1* genotype of subjects from SKS. Among the 465 participants who had samples available for analysis, 23.4% self-described their race as Black, 59.6% as White, and 17.0% as Other (**Table B in S1 File**). Similar to what was previously demonstrated [15], the plasma concentration of APOL1 in Black participants was significantly higher than in White participants [median (IQR): 370.7 nM (307.0-483.8) vs. 250.7 nM (200.4–314.5), *P* < 0.0001, Wilcoxon test; **Table C in S1 File**], which was independent of eGFR, urine albumin-to-creatinine ratio, and serum total protein concentration. In contrast to previous studies [15, 30], there was a weak correlation between eGFR and the APOL1 plasma concentration in the entire cohort (**Fig 1A**; r = 0.11; *P* = 0.013), which was also statistically significant in the subcohort of Black participants (N = 109, r = 0.22, *P* = 0.025). Importantly, the total concentration of APOL1 in plasma (i.e., the concentration of all isoforms) was not statistically different between Black participants with risk variant *APOL1* and those with *APOL1*-G0 (**Fig 1B**).

### Circulating risk variant APOL1 is biochemically different from APOL1-G0

In contrast to what was observed in bulk plasma, the relative amount of APOL1 in the HDL fraction of plasma was significantly lower in Black participants who carried risk variant *APOL1* alleles compared with those who did not (**Fig 1C**), which suggested that most of the risk variant APOL1 resides in a different fraction of plasma, *i*.*e*., other than the HDL fraction (density = 1.063–1.210 g/mL) that is traditionally purified by density gradient ultracentrifugation. In participants who were heterozygous for risk variant *APOL1*, we demonstrated that APOL1-G0 peptide was under-recovered in the HDL fraction of plasma compared with bulk plasma (**Fig 1D**), which might suggest that risk variant APOL1 protein shifts APOL1-G0 protein to another fraction of plasma via heterodimer formation, but this is speculative. In support of this hypothesis, asymmetric flow field-flow fractionation demonstrated that APOL1 resided in larger particles in heterozygous individuals compared with homozygous *APOL1-G0* participants (**Fig 1E**). Similar trends (lower concentrations of APOL1 protein in 9–11 nm diameter particles) were observed in the subgroup of Black participants, including those who were homozygous for risk variant *APOL1* (**Fig B in S1 File**). The size of the larger particles appeared to be similar in size to apolipoprotein E-containing particles (**Fig C in S1 File**).

### Urine concentrations of APOL1 are associated with plasma APOL1 concentrations and eGFR

The differences observed in the biochemical characteristics of polymorphic APOL1 in plasma raised the possibility of a dynamic process of APOL1 movement between particles and the possibility that APOL1 could be filtered into the urine (i.e., like albumin) and contribute to renal cell damage. To test this hypothesis, we determined the concentration of APOL1 in urine samples available from participants enrolled in SKS. The concentration of APOL1 in urine was significantly lower than in plasma (median [IQR], 49.9 [14.2–148.4] pM vs. 279.6 [217.1–352.0] nM, respectively). There was a modest positive correlation between plasma APOL1 concentration and urine APOL1 concentration (**Table D in S1 File**, r = 0.01, *P* = 0.03) and a weak inverse correlation between urine APOL1 concentration and eGFR (**Fig 1F, Table D in S1 File**; r = -0.27; *P* < 0. 001), which was independent of self-described race and *APOL1* genotype. In other univariate analyses, the amount of APOL1 in urine was positively correlated with plasma cholesterol concentration and urine albumin-to-creatinine ratio (**Table D in S1 File**; r = 0.02, *P* = 0.003 and r = 0.22, *P* < 0. 001, respectively). Importantly, in a multivariable model, the concentration of APOL1 in urine remained inversely correlated with eGFR, independent of urine albumin-to-creatinine ratio (**Table D in S1 File**, *P* = <0.001). It should be noted that while there was a positive correlation between plasma APOL1 concentration and eGFR and a positive correlation between plasma APOL1 and urine APOL1 concentrations, there was a negative correlation between urine APOL1 concentration and eGFR. This is likely explained by the weak correlation between urine and plasma APOL1 concentrations. Together, these data support a hypothesis in which circulating APOL1 enters the urine similar to albumin and may play a role in the development and progression of kidney disease. However, it is important to point out that these data are cross-sectional and repeated measures in the same human participants would be needed to further support the hypothesis that APOL1 protein enters the urine due to incident injury.

### Alb/APOL1 mice express APOL1 RNA in liver and human APOL1 protein circulates in plasma

**Fig 2:**
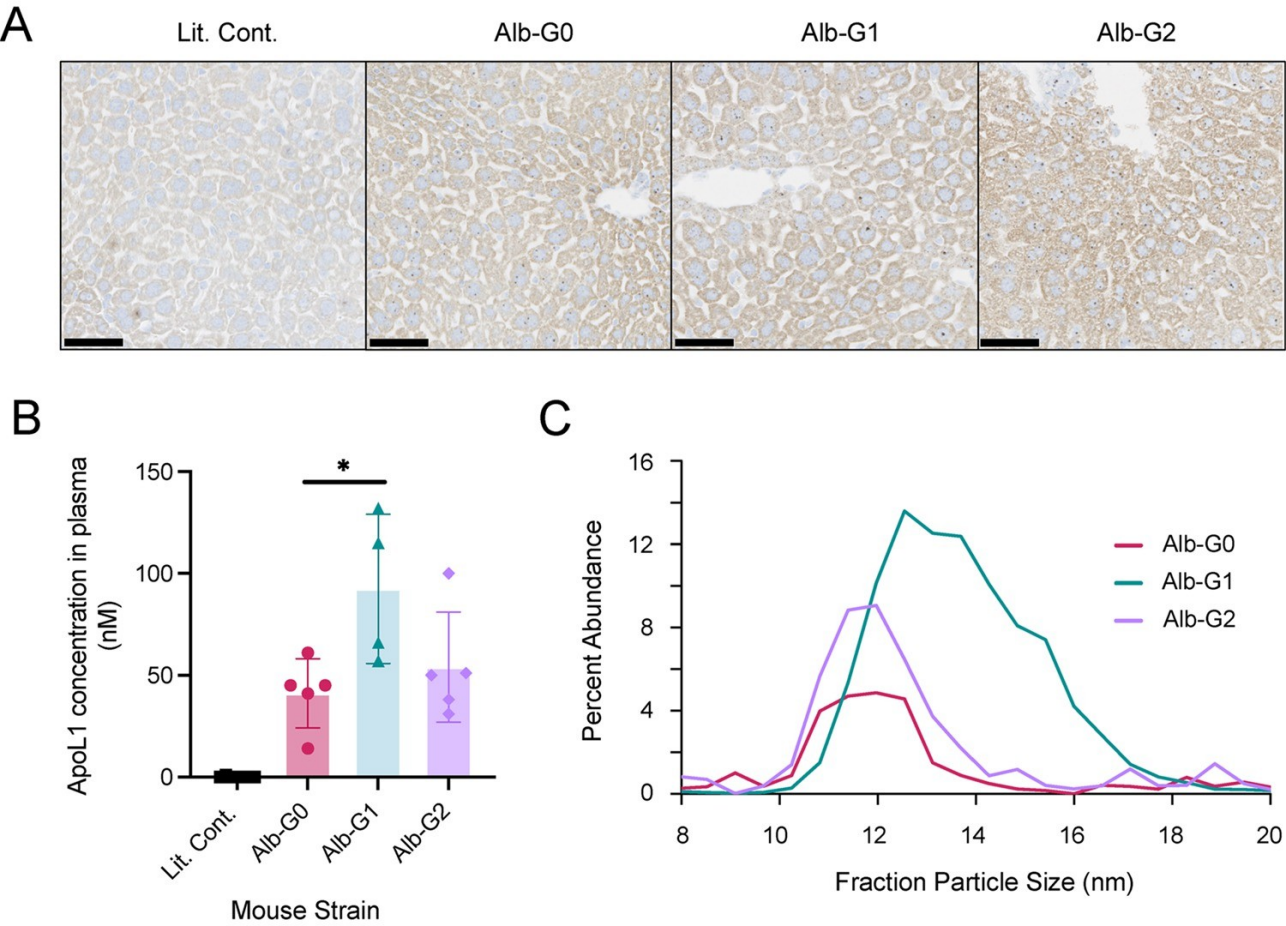
A novel transgenic mouse model. (A) *In situ* hybridization was used to detect *APOL1* expression in the livers of mice expressing different alleles of *APOL1* from a murine albumin promoter (Alb-G0, Alb-G1, and Alb-G2). Brown punctate staining represents detection of *APOL1* RNA within hepatocytes. Scale bars are 50 μm. (B) APOL1 expressed in the liver circulates in plasma. Circulating human APOL1 was quantified in mouse plasma after the triple intervention by using a targeted LC-MS/MS assay. Data are displayed as mean ± SD. Statistical significance was determined using Student’s t-test. *P<0.05. (C) Compared with APOL1-G0, risk variant APOL1 resides on larger lipoprotein particles in mice. Asymmetric-flow field-flow fractionation of Alb/APOL1 mouse plasma demonstrated that APOL1-G1 is shifted to larger particles compared to APOL1-G0, similar to what is observed in human plasma. The effect was less substantial in Alb/APOL1-G2 mice.

To more directly test the hypothesis that circulating APOL1 could exacerbate kidney injury, we developed a murine model that expresses *APOL1* only in liver. By *in situ* hybridization, *APOL1* RNA was detected in the livers of transgenic mice expressing *APOL1*-G0, *APOL1*-G1, or *APOL1*-G2 from an albumin promoter (**Fig 2A**). We confirmed that APOL1 was expressed in liver, but not in kidney or spleen (**Fig D in S1 File**). Human APOL1 protein was present in transgenic mouse plasma at concentrations near to or lower than those found in human plasma (10–130 vs. 87–732 nM, respectively), suggesting that the Alb/APOL1 mouse could be a suitable model to characterize the effects of plasma risk variant APOL1 protein *in vivo* (**Fig 2B**). The plasma concentration of APOL1 in Alb/APOL1-G1 mice was statistically higher than Alb/APOL1-G0 mice and similar with and without triple intervention (**Fig E in S1 File**). Asymmetric flow field-flow fractionation demonstrated that the APOL1-G1 variant protein was preferentially carried on larger particles, as it is in human plasma, suggesting that the variant amino acid changes impart the different biochemical behavior of risk-variant APOL1 protein seen in human plasma (**Fig 2C**). The APOL1-G2 protein was shifted less substantially to larger particles.

### Circulating risk variant APOL1 exacerbates kidney injury in a mouse model

**Fig 3:**
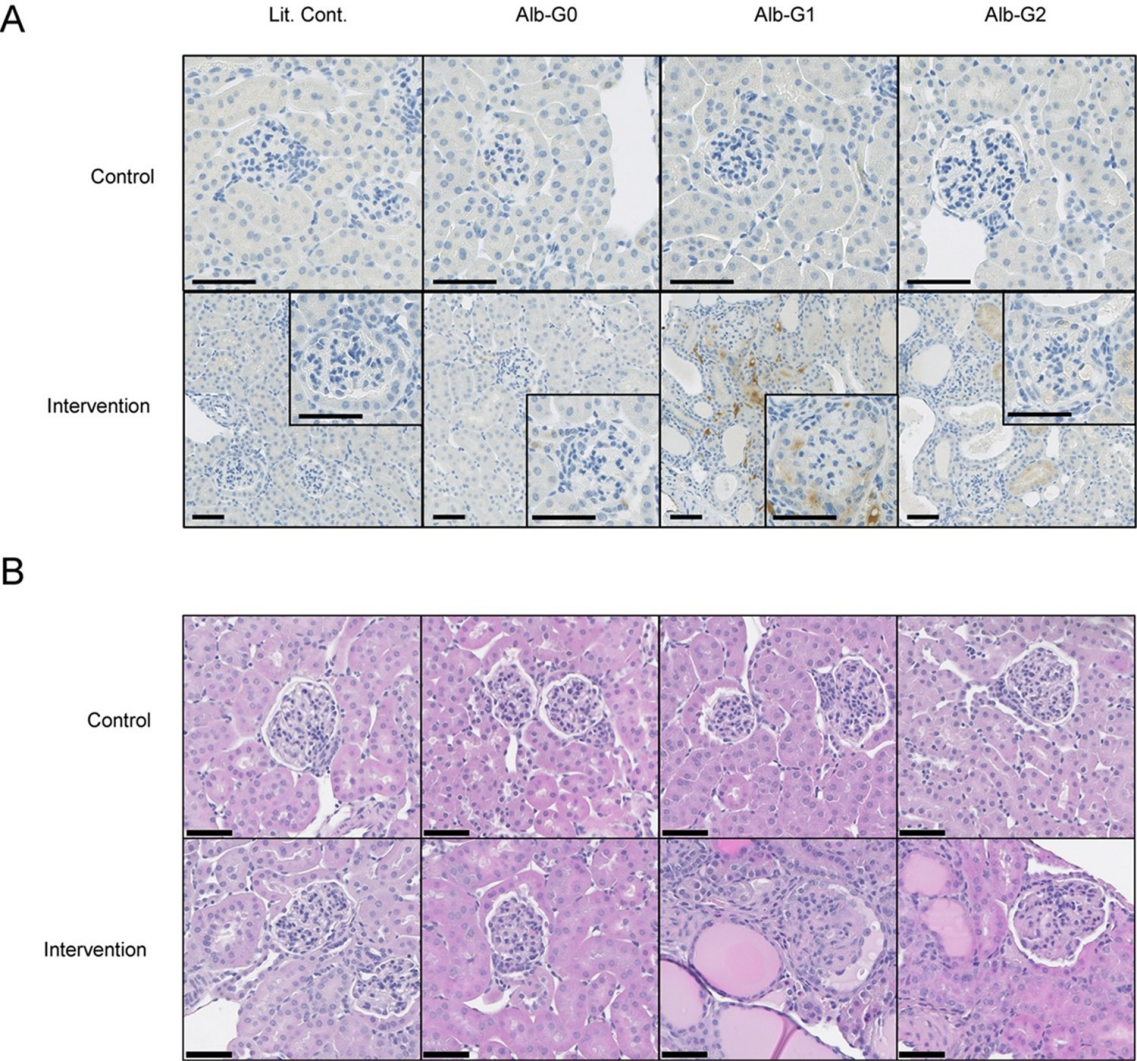
Circulating risk variant APOL1 and pathological changes. (A) Circulating APOL1 protein localizes to injured kidneys. To induce kidney damage and proteinuria in Alb/APOL1 mice (Alb-G0, Alb-G1, and Alb-G2) and their non-transgenic littermate controls (Lit. Cont.), mice received interferon-γ, puromycin, and basic fibroblast growth factor. Immunohistochemical detection of APOL1 protein in kidney from Alb/APOL1 mice after kidney-damaging intervention is shown compared with their littermate controls and non-treated control animals. Risk variant APOL1 protein localized to the kidney in Alb/APOL1-G1 mice and to a lesser extent in Alb/APOL1-G2 mice. Scale bars are 25 μm. (B) Circulating risk variant APOL1 is associated with pathological changes in the triple intervention model. Hematoxylin and eosin staining of kidney from Alb/APOL1 mice with and without the triple intervention demonstrated pathological differences in kidneys from treated mice that have circulating APOL1-G1 and APOL1-G2. Scale bars are 50 μm.

**Fig 4:**
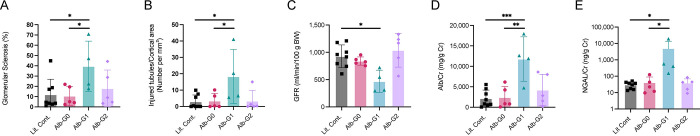
Circulating risk variant APOL1 causes kidney dysfunction and tubular damage. (A, B) Transgenic Alb/APOL1-G1 mice had a significantly higher amount of glomerular sclerosis and tubular injury. Kidneys were evaluated after being stained with periodic acid-Schiff stain. (C) Transgenic Alb/APOL1-G1 mice demonstrated reduced measured glomerular filtration rate (GFR) at day 10. Clearance of intravenously or retro-orbitally administered FITC-sinistrin was used to quantify glomerular filtration in mice on day 10 of the triple intervention. (D, E) Transgenic Alb/APOL1-G1 mice have higher albumin-to-creatinine ratio (Alb/Cr) and NGAL-to-creatinine ratio (NGAL/Cr). Urine concentrations of albumin, NGAL, and creatinine were determined on day 14 of triple intervention therapy. Data are presented as mean ± SD. Statistical significance was determined by Student’s t-test. *P<0.05, **P<0.01, ***P<0.001.

**Fig 5:**
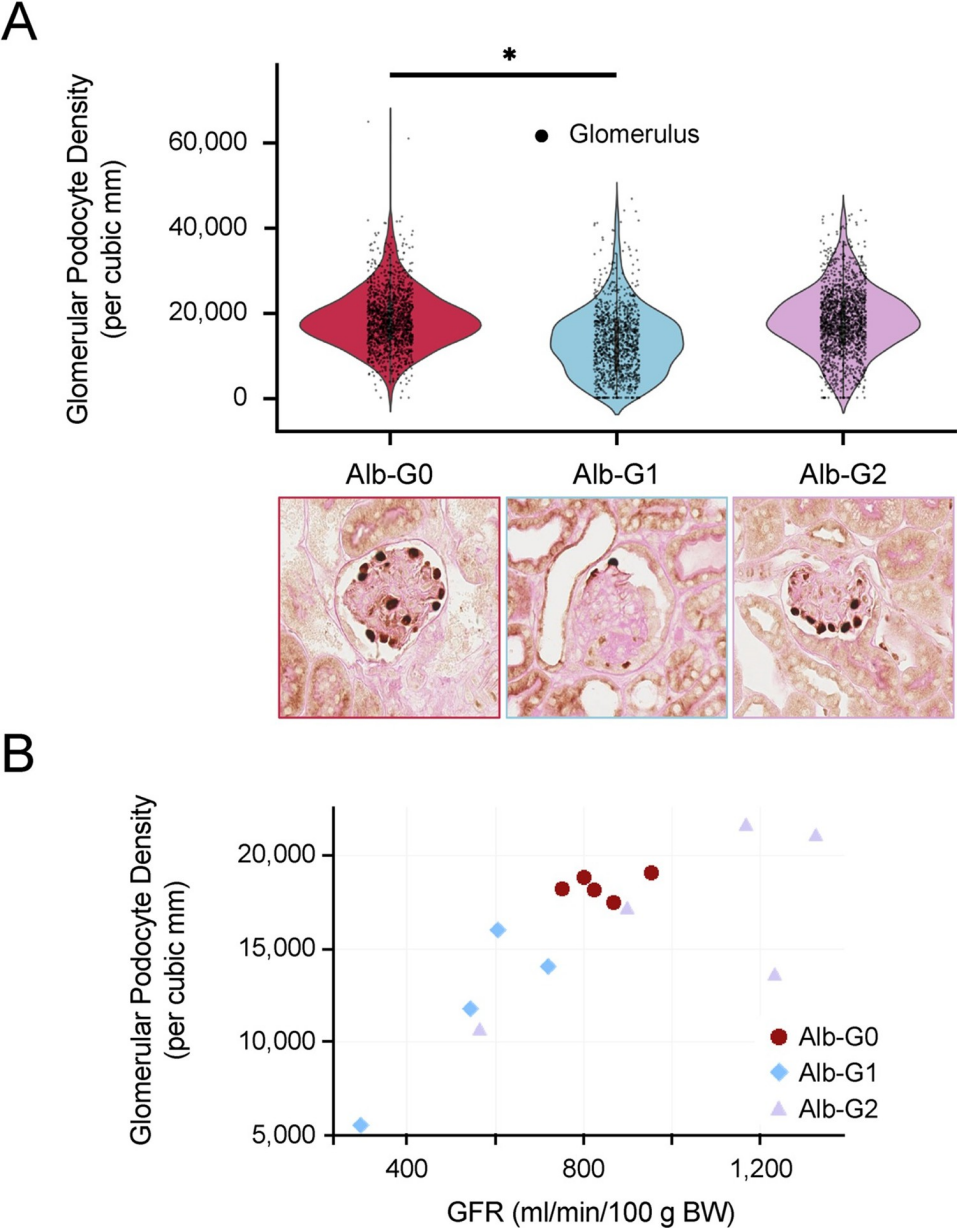
Circulating risk variant APOL1 causes podocyte loss. (A) Podocyte density is lower in glomeruli from Alb/APOL1-G1 mice. Podocount, a computational tool for whole-slide podocyte estimation, was used to derive podometrics from digitized kidney slides. Podocyte density for each glomerulus was computed as the ratio of podocyte count to glomerular cross-sectional area and is plotted for each genotype. Data are shown as violin plots for each Alb/APOL1 genotype. Superimposed points represent individual glomeruli. Statistical significance was determined by Student’s t-test. *P<0.05. (B) Loss of podocyte density is correlated with kidney dysfunction. Glomerular podocyte density was compared with the GFR measured at day 10 of the intervention and demonstrated a significant association between podocyte density and kidney function. The Pearson correlation coefficient (R) for the association was 0.742 and the *P*-value determined from linear regression was < 0.001.

As described in previous publications [26, 36], kidney injury was initiated in Alb/APOL1 transgenic mice using a triple intervention approach (interferon-γ, puromycin, and basic fibroblast growth factor), which targets the podocyte. In the absence of the triple intervention and kidney damage, APOL1 protein was not detected in glomeruli by immunohistochemistry; however, APOL1 protein was detected in the glomeruli of Alb/APOL1-G1 transgenic mice on day 14 of the intervention (**Fig 3A**). APOL1 protein appeared in the vascular lumen and within proximal tubular cells in Alb/APOL1-G1 mice, which may reflect non-specific uptake of APOL1 that has entered the tubular lumens. However, it was not clear from these images that APOL1was present in the glomerular filtrate in Alb/APOL1 mice. After the triple intervention, there were pathological changes consistent with kidney damage (*e*.*g*., glomerular sclerosis, microcystic tubular dilatation, and tubular atrophy) (**Fig 3B**), which were significantly more common in the Alb/APOL1-G1 mice compared to Alb/APOL1-G0 mice and their littermate controls (**Fig 4A and 4B**). Kidney dysfunction, as assessed by reduced GFR and increased albuminuria, was present in Alb/APOL1-G1 mice after intervention (**Fig 4C and 4D**). Tubular injury, as measured by urinary NGAL, was most pronounced in Alb/APOL1-G1 mice (**Fig 4E**). Podocyte density was reduced in Alb/APOL1-G1 mice compared with Alb/APOL1-G0 and Alb/APOL1-G2 mice (**Fig 5A**). Further, the extent of podocyte loss in glomeruli correlated with albuminuria (**Fig 5B**). Taken together, data from this mouse model support the hypothesis that circulating risk variant APOL1 can exacerbate kidney injury *in vivo*.

## Discussion

While we are unable to exclude an important role of podocyte-derived APOL1 in the progression of kidney injury, our data provide new evidence that liver-derived risk variant APOL1 circulating in plasma could contribute to the exacerbation of kidney injury in patients who are homozygous or compound heterozygous for risk variant alleles. The different biochemical properties of the risk variants, which lead to a shift of APOL1 protein out of the classically defined HDL fraction of plasma and into larger particles (Fig 1), complements previous studies demonstrating that risk variant APOL1 interacts with membranes and lipid droplets differently from APOL1-G0 [10, 25, 28, 31, 48]. These differences may explain the increased levels of endoplasmic reticulum stress [28], mitochondrial instability [27], and inflammasome activation [6, 25] that are observed in cell culture experiments using variant APOL1. The differences may also have important clinical implications with respect to the ability of APOL1 binding to the trypanosome serum resistance antigen protein (which inactivates APOL1-G0), to kill trypanosomes *in vivo*, or minimize symptoms in infected individuals [49–51]. The difference in particle size does not appear to be related to a shift of APOL1 protein between previously described trypanosome lytic factors (TLF1 and TLF2), because mice lack haptoglobin related protein, which is an important component of the TLFs [52].

**Fig 6:**
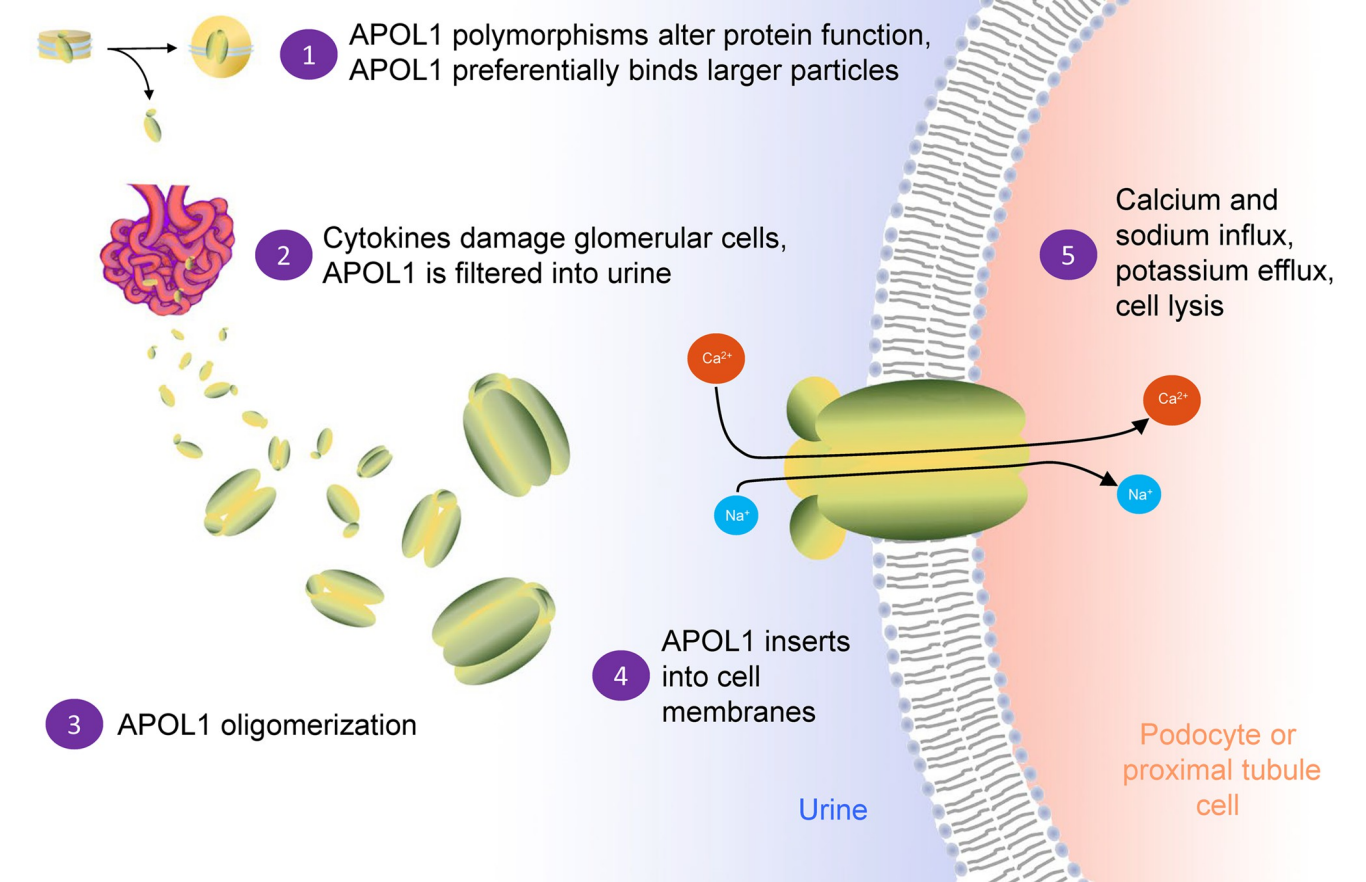
A speculative model for the role of circulating APOL1 in kidney injury. High-risk coding variants have different biochemical characteristics in plasma compared with *APOL1*-G0 and are associated with larger particles. Leakage of APOL1 into Bowman’s space in a manner similar to albumin allows for the formation of APOL1 multimers that can insert into the plasma membrane of podocytes or proximal tubule cells once the pH of the glomerular filtrate becomes acidic. Calcium and sodium influx and subsequent potassium efflux leads to proximal tubule cell death and exacerbated kidney injury.

Previous studies have demonstrated that plasma-derived APOL1 enters the urine under normal conditions [53]. Given the correlation of albumin and APOL1 concentrations in human urine (Table D in S1 File), the movement of APOL1 from plasma to urine may be similar to that of albumin. Local inflammation and protein leakage through the glomerulus could theoretically provide a possible mechanism that could accentuate this process, giving plasma APOL1 better access to podocytes and proximal tubules (a speculative model is shown in Fig 6). In addition, urine provides an acidic environment, with luminal pH of 6.9 in the proximal tubule and 6.7 in the distal tubule [54]. This would favor plasma membrane insertion and lead to the formation of an active homotetrameric pore complex that could cause intracellular potassium loss, calcium influx, and cell lysis [21, 22, 28]. This cell death would be manifested as the observed human pathology of microcystic tubular dilatation and tubular atrophy with glomerulosclerosis and collapsing glomerulopathy [55]. Kidney diseases that are known to be associated with APOL1-driven pathology, including HIV-associated nephropathy, focal segmental glomerulosclerosis, and lupus nephritis, have been modeled in transgenic mice that express human *APOL1* by several approaches [7, 56]. Cytokine-induced disruption of podocyte function, loss of glomerular basement membrane integrity, and reduced ability to retain proteins the size of APOL1, with its predicted molecular weight of 42 kDa, could be the prelude to injury of vulnerable cells by risk variant APOL1 in the glomerular filtrate, even in heterozygous individuals, as observed in patients with HIV-associated nephropathy [57].

Up to now, almost all mouse experiments have used models that express APOL1 in the podocyte [50, 58–60]. The results from the novel mouse model described here provide some support for the hypothesis that plasma APOL1, particularly APOL1-G1, could synergize with APOL1 protein expressed in podocytes to exacerbate renal injury. Although some pathological changes were also observed in the Alb/APOL1-G2 mice after the triple intervention, there were no measurable differences in glomerular sclerosis, tubular injury, GFR, or albuminuria. This finding, that Alb/APOL1-G1 mice manifested significant renal injury within two weeks while Alb/APOL1-G2 mice did not, suggests functional differences between APOL1-G1 and APOL1-G2 in plasma, as has been suggested previously [50, 61–64]. It may also be consistent with what is observed in human populations, in which *APOL1-G1* associates with more significant albuminuria and kidney disease compared with *APOL1-G2* in homozygous individuals [65–67], and with HIV-associated nephropathy and hypertension-associated end stage kidney disease in heterozygous individuals [57, 68]. However, it is also possible that longer observation might have been needed to observe changes in GFR or urine albumin-to-creatinine ratio in Alb/APOL1-G2 mice. Future studies might examine whether the effects of APOL1 variants in plasma extend to other mouse models of kidney injury and whether mouse plasma with APOL1 renal risk variants or purified APOL1 protein, when administered to other mice, can also induce kidney injury.

In addition to providing supporting evidence for a new, complementary mechanism to kidney disease progression, our results may also be important for the field of renal transplantation. Soon after the discovery that *APOL1* polymorphisms account for a substantial fraction of the increased risk of kidney disease in people of sub-Saharan African descent [1, 2], there were reports that the *APOL1* genotype of kidney donors [14], but not transplant recipients [13], was strongly associated with worse allograft outcomes and donated organs with two *APOL1* risk variant alleles have shorter survival compared to organs with one or no risk variant alleles. These reports have been corroborated in several subsequent studies [12, 69–72] and there has been a proposal to incorporate genetic testing into kidney donation programs moving forward [73]. More recently, there have been two studies that have raised awareness of the potential role of recipient *APOL1* genotype as well in transplant outcomes [16, 74, 75]. Our current findings in human plasma and human urine, which are correlative and hypothesis-generating, as well as our data from *APOL1* transgenic mice support a possible role for recipient APOL1 protein in the kidney disease process. The roles of donor and recipient genotypes are being prospectively investigated in the APOLLO study [76].

### Limitations

While our study has notable strengths, including a large, well-characterized clinical cohort at various stages of chronic kidney disease, state-of-the-art methods to characterize lipoprotein particles in plasma, and a novel mouse model to evaluate the effects of circulating liver-derived APOL1 on disease progression, it has numerous limitations. First, the human data are cross-sectional and correlative and cannot be used to identify mechanisms or causality. As a result, these data instead lead to a compelling hypothesis that will need additional experiments, including mechanistic studies *in vitro*, to gain a full understanding of the relationships that we have identified. Second, the concentrations of APOL1 circulating in mouse plasma were different for each *APOL1* polymorphism, with Alb/APOL1-G1 mice having significantly higher concentrations of APOL1 than Alb/APOL1-G0 or Alb/APOL1-G2 mice. This complicates the comparison of findings between mice expressing APOL1 with different polymorphisms, particularly because the higher APOL1 concentrations in Alb/APOL1-G1 mice were associated with the higher levels of glomerulosclerosis, tubular injury, and albuminuria, as well as reduced eGFR. However, because the concentration of APOL1 in Alb/APOL1-G1 mice was generally lower than what is observed in humans, our data do support the hypothesis that circulating APOL1-G1 protein can exacerbate kidney injury, as evidenced by the comparison of Alb/APOL1-G1 mice with Alb/APOL1-G0 mice or their littermate controls. Third, the concentration of urine albumin in our mouse model was much higher than that observed in the human cohort, which suggests that the kidney injury in our mouse model was much more severe and thus less generalizable to human disease. Fourth, mice lack haptoglobin related protein, which is an important constituent in trypanosome lytic factor in humans, further limiting our ability to generalize our mouse model to human disease. Previous studies by the Raper laboratory have demonstrated that when haptoglobin related protein is expressed in mice along with APOL1, the resulting APOL1-containing lipoprotein particles are different than when APOL1 is expressed alone [77]. It would therefore be interesting to determine the effects of haptoglobin expression on our Alb/APOL1 model. Lastly, there is a possibility that the kidney damage observed in the Alb/APOL1-G1 transgenic mice could be due to insertional mutagenesis in an unrelated gene. However, since the tubular injury phenotype observed in our Alb/APOL1-G1 mouse model resembles that seen in human patients, we think it is more likely that the tubular injury is due to circulating APOL1.

## Conclusions

Given these important limitations, our human and mouse studies are not conclusive. However, our data do support a hypothesis in which circulating APOL1 contributes to the renal dysfunction and pathology seen in humans. The leakage of APOL1 into urine, pore assembly in an acidic environment, and insertion into cell membranes could theoretically damage proximal tubules and contribute to the loss of kidney function. In addition, if plasma APOL1 plays a role in kidney disease, then it will be important to develop pharmaceuticals that prevent plasma and urine APOL1 multimerization.

## Supporting information

S1 File (PDF)

## Abbreviations

AF4: asymmetric flow field-flow fractionation
APOL1: apolipoprotein L1
CKD: chronic kidney disease
eGFR: estimated glomerular filtration rate
GFR: glomerular filtration rate
SKS: Seattle Kidney Study
