# Supplementary material for: Variant APOL1 protein in plasma associates with larger particles in humans and mouse models of kidney injury
Source: PLoS One. 2022 Oct 24;17(10):e0276649. doi: 10.1371/journal.pone.0276649 (PMC9591058; doi:10.1371/journal.pone.0276649)
Supplement: S1 File — (PDF) [file pone.0276649.s001.pdf]

Variant APOL1 protein in plasma associates with larger particles in humans and mouse models of kidney injury

Michael Andrews, Teruhiko Yoshida, Clark M. Henderson, Hannah Pflaum,
Ayako McGregor, Josh Lieberman, Ian H. de Boer, Tomas Vaisar,
Jonathan Himmelfarb, Bryan Kestenbaum, Joon-Yong Chung, Stephen M. Hewitt,
Briana A. Santo, Brandon Ginley, Pinaki Sarder, Avi Z. Rosenberg,
Taichi Murakami, Jeffrey B. Kopp, Zsuzsanna Kuklenyik, and Andrew N. Hoofnagle

Supplemental Material Table of Contents

Supplemental Methods. 2

Supplemental Table A.
Transitions monitored for the quantification of total APOL1 in plasma. 12

Supplemental Table B.
Participant characteristics in the Seattle Kidney Study cohort. 13

Supplemental Table C.
Association of self-described race on APOL1 protein concentration in G0/G0 participants. 14

Supplemental Table D.
Associations between APOL1 concentration in urine with clinical variables. 15

Supplemental Figure A.
Measured hydrodynamic diameter as function of fraction number. 16

Supplemental Figure B.
Lipoprotein particle size distribution in Black participants. 17

Supplemental Figure C.
Apolipoprotein size distribution determined by asymmetric flow field-flow fractionation. 18

Supplemental Figure D.
Fluorescent in situ hybridization in liver, kidney, and spleen. 19

Supplemental Figure E.
Concentration of APOL1 protein in mouse plasma before and after the triple intervention. 20

Supplemental Methods

Human samples

Plasma, urine, and DNA samples were obtained from participants in the Seattle Kidney Study (**SKS**), an IRB-approved cohort study described previously [1]. All participants provided written informed consent. Participants self-reported their race as Black, White, Asian, American Indian, or other. They separately reported Hispanic ethnicity (yes versus no). For this analysis, we defined Black race as all persons who self-reported their race as Black regardless of Hispanic ethnicity. eGFR was calculated from plasma creatinine using the 2021 CKD-EPI equation without a race coefficient included [2], and urine albumin-to-creatinine ratios were determined using an automated clinical chemistry analyzer (Beckman DxC 800, Beckman Coulter, Brea, CA). Amounts of APOL1 in the HDL fraction of plasma were determined previously.[3] Sufficient plasma to quantify APOL1 concentrations was available from 465 participants. Of these, 98 self-identified Black participants had enough DNA for *APOL1* genotyping, 382 participants had sufficient urine from the same time-point as the plasma to quantify APOL1 in urine, and 319 participants had data available for all covariates (including eGFR) for multivariate linear regression to evaluate associations with urinary concentrations of APOL1. In addition to samples from SKS participants, four fresh human plasma samples were acquired from Interstate Blood Bank (Memphis, TN) and homogenized using equal volumes to prepare calibration and quality control materials for size fractionation experiments. After mixing, these quality control materials and calibrators were dispensed into 15 µL and 235 µL aliquots, respectively, and stored at -80°C until further analysis. De-identified SKS samples were received and analyzed at the Centers for Disease Control, where the study was approved as research not involving identifiable human subjects under U.S. Health and Human Services Department Policy for Protection of Human Research Subjects codified in the Code of Federal Regulations at 45 CFR part 46.

Quantification of total APOL1 in human plasma

Plasma samples were reduced and denatured by adding 5 µL plasma to each well of a 96-well 2 mL deep-well plate containing 31 µL ammonium bicarbonate (100 mM), 1 µL dithiothreitol (0.5 M), and 4 µL heavy-labeled APOL1 internal standard peptides (New England Peptide, Gardner, MA, 3 µM). Then, 35 µL trifluoroethanol was added to each sample, mixed on a multi-tube vortexer for 10 seconds at speed 7, and mixed for 1 h at 1,400 rpm and 65°C in an

Eppendorf ThermoMixer C (Enfield, CT). To alkylate the samples, 4 µL iodoacetamide (0.5 M) was added to each well, vortexed on a multi-tube vortexer, and incubated in the dark for 30 min at room temperature. Samples were diluted to < 5% v/v trifluoroethanol with 600 µL of 38.6 mM ammonium bicarbonate/1.6 mM dithiothreitol (quenching the remaining iodoacetamide) and mixed on speed 6 for 10 s on a multi-tube vortexer. Next, to digest the samples, 18.5 µL trypsin (2 mg/mL) was added to each sample and mixed for 30 min at 1,400 rpm and 37°C in a ThermoMixer. To stop the digestion, 3 µL 88% formic acid was added to each sample followed by mixing. Lastly, 10 µL of the sample digest was injected onto the LC-MS/MS instrument.

LC-MS/MS analysis for the quantification and phenotyping of total APOL1 in plasma

Chromatography was performed on a Waters Acquity I-Class system (Milford, MA), equipped with a flow-through needle, a Waters HSS 2.1x50 mm 1.8 µm T3 modified C18 column, and an associated pre-column at 50°C and at a flow rate of 0.3 mL/min. The 5.5 min linear liquid chromatography started at 90% A (0.1% formic acid in water)/10% B (0.1% formic acid in methanol) and ended at 30% A/70% B. The column was cleaned with 70% B for 1 min at 0.6 mL/min, then re-equilibrated at 98% A for 1.8 min at 0.6 mL/min flow rate, then at 90% A for 0.1 minute at 0.3 mL/min. The sample compartment was held at 5°C during analysis. Data acquisition was performed with a Waters TQ-S micro with electrospray ionization in MS/MS mode. The source was operated at 150°C with a capillary voltage of 3 kV, cone gas flow at 200 L/h and desolvation gas flow at 1,000 L/h. The transitions used for each of the peptides monitored, as well as the instrument-specific settings for each peptide, are listed in **Supplemental Table A**.

APOL1 genotyping

The *APOL1* G0, G1 (rs73885319), and G2 (rs71785313) alleles were detected by multiplexed qPCR with TaqMan allele-specific primer/probe genotyping assays (ThermoFisher Scientific, Waltham, MA, Cat No. 4351379). We used a commercially available set of primers and probes that distinguish the G0 and G1 alleles (ThermoFisher Assay ID: C__98253221_10). To distinguish G0 from G2 alleles, a custom genotyping assay (Assay ID: AN7DPWD) was designed using ThermoFisher's custom TaqMan SNP assay design tool following the manufacturer's instructions resulting in the following primers and probes: Fwd primer 5'-GCTCAGGAGCTGGAGGAGAA-3', Rev primer 5'-CCTGCCCTGTGGTCACA-3', G0-

specific probe 5'-CCTGCAGAATCTTATAATT-3', G2-specific probe 5'-CCTGCAGAATCTTATTG-3'. All reactions were performed in technical duplicate with 6 ng template genomic DNA, with amplification according to the manufacturer's instructions using the TaqMan Genotyping Master Mix containing AmpliTaq Gold polymerase (Cat No. 4371357) and annealing temperature of 62°C. All PCR was performed on the ViiA 7 Real-Time PCR System (ThermoFisher Scientific), and analyzed by using the accompanying ViiA software for SNP classification with three HapMap control cell lines of known genotype at the *APOL1* locus purchased from Coriell Institute (Camden, NJ): G0/G0, cell line NA12878; G0/G1, cell line NA20362; and G0/G2, cell line NA20348.

APOL1 phenotyping

The isoforms of APOL1 present in each plasma sample were determined by LC-MS/MS by comparing the ratio of each polymorphic peptide (LNILNNNYK or LNMLNNNYK) to a non-polymorphic peptide (VTEPISAESGEQVER) with empirical minimum cutoffs to detect the presence of peptide vs. absence of peptide, and therefore each haplotype. From these cut-offs, the *APOL1* genotype could be identified for each participant. The phenotype approach (used in N = 465 participants) was cross-validated with the results of the genotyping approach that was performed in a subset of self-identified Black participants (N = 98 total; 33 G0/G0, 27 G0/G1, 10 G1/G1, 4 G1/G2, and 4 G2/G2), which demonstrated a 100% agreement between the genotype and the observed phenotype.

Size fractionation

Plasma samples were thawed in sets of six and injected into the asymmetric flow field-flow fractionation (**AF4**) system chamber using an autosampler, which was maintained at 4°C during analysis. The AF4 system (AF2000, PostNova Analytics, Salt Lake City, UT) operated with a buffer carrier fluid composed of 10 mM sodium bicarbonate/150 mM sodium chloride (pH 7.4) in deionized water with an outlet flow of 0.1 mL/min. A UV detector monitored the reproducible retention times of dominant plasma proteins. From 50 µL plasma injected into the AF4 system, 40 fractions were collected in 2.5 min time increments. During elution, the flow rate was recorded by a microfluidic flow meter (Elveflow, Paris, France), placed between the UV detector and the fraction collector. Each fraction was collected into 1 mL deep-well 96-well Eppendorf plate by an Agilent 1200 fraction collector (Santa Clara, CA), chilled at 4°C. The exact volume

of each fraction (~300 µL) was calculated from the collection-time increments and the corresponding flow rates readings on the microfluidic flow meter. The exact volume was used for the calculation of the amount of protein in the entire fraction, dividing by the 50 µL plasma injected into the AF4 system yielded protein concentrations in plasma.

Dynamic light scattering

For each of the 40 fractions, a 20 µL aliquot was transferred to a clear-bottom 384-well plate (Sigma-Aldrich, St. Louis, MO) with a liquid handling robot (Beckman Coulter, Indianapolis, IN). The average hydrodynamic size of particles in each fraction was determined by a dynamic light scattering using a Dynapro plate reader (Wyatt Technologies, Santa Barbara, CA). Certified National Institute of Standards and Technology Traceable Particle Size (20-100 nm) Standards (ThermoFisher Scientific) were used to validate the accuracy of the dynamic light scattering (DLS) measurements. In some fractions, where the low concentration of particles did not allow accurate size measurements, hydrodynamic size was determined by interpolation using a size vs. fraction number empirical quadratic function determined based on the high concentration fractions (**Supplemental Figure A**).

Protein analysis in AF4 fractions

For the LC-MS/MS analysis of proteins in the AF4 fractions, a 50 µL aliquot of each fraction was diluted 3-fold with buffer at pH 7.40 containing 10 mM sodium bicarbonate/150 mM sodium chloride/0.15% Zwittergent 3-12 (EMD Millipore, Billerica, MA). The analysis was performed with an on-line column switching system (Shimadzu Scientific Instruments/Perfinity Biosciences, West Lafayette, IN), equipped with a trypsin column (2.1 x 50 mm), a trapping column (HALO C18 core shell 5 x 2.1 mm, 2.7 µm particle size), and an analytical column (HALO C18 core shell 100 x 2.1 mm, 2.7 µm particle size). The autosampler was programmed to pick up 50 µL from each diluted fraction and 5 µL isotopically labeled peptide mix (~10 pmol/mL) from a separate vial. Merged in the autosampler loop, the sample and the labeled peptides were passed through the on-line trypsin column at 25 µL/min. Peptides were collected on the trapping column for 8 min and then transferred to and resolved with the analytical column using a 3-95% acetonitrile/0.1% formic acid/water gradient at 350 µL/min (total run time with column equilibration was 12 min). Peptide transitions were monitored using a 6500 QTRAP (AB Sciex, Foster City, CA, USA) in multiple reaction monitoring mode (**Supplemental Table A**).

Although 30 proteins were monitored in each size fraction, here we only present data for APOL1 in the main text, and for apolipoprotein A-I and apolipoprotein E in supplemental information. The peptides used for quantification of total APOL1 in plasma (VTEPISAESGEQVER and ALDNLAR) were not detected using these digestion conditions, except in mice, where the ALDNLAR peptide was detectable.

Deconvolution of size profiles

Using JMP software (SAS Institute, Cary, North Carolina), the experimental concentration vs. hydrodynamic size profiles were deconvolved using the following formula

$$f(\Theta, d, s, \Delta, w, n) = \sum_{i=1}^{n} \Theta_i * e^{-\frac{(d-s+i\Delta)^2}{2w^2}} \qquad [1]$$

where d was the measured size in nm for each fraction (from dynamic light scattering), i was the number of increments from point s, Δ is the size of one increment, and w is the ½ peak width. Because the cumulative value of the exponential terms is 1, only the Θ_i variables in Equation 1 affects the intensity of the Gaussian components. With constant s=6 nm, Δ=1 nm and w=0.5 nm, for n=9 (nine Gaussian components), the Θ_i values were optimized in maximum 500 iteration cycles or until $f(d)$ fitted the experimental size profile for each protein. The optimized Θ_i values were used for the calculation of the individual Gaussian peaks, allowing to show them in overlay with the corresponding experimental profiles. The de-convoluted data could be represented simply by the Θ_i values, and the percent abundance of each Gaussian component calculated by

$$\%Abundance = \frac{\Theta_i}{\sum_{i=1}^{n} \Theta_i} \qquad [2]$$

Quantification of APOL1 in human urine

Proteins in urine were precipitated by combining 200 µL well-mixed urine, 10 µL *Aspergillus oryzae* α-amylase (0.2 µg/µL, as a bulking agent), and 800 µL ice-cold acetone/1 mM hydrochloric acid in a 1.5 mL Eppendorf Lo-Bind Snap Lock tube, vortexed, and incubated at -20°C for 24 h. Cold mixtures were centrifuged for 20 min at 4°C and 15,000x*g* and the supernatant was decanted and discarded. The pellets were dried-down in a Labconco CentriVap SpeedVac (Kansas City, MO) at 25°C until dry. Precipitates were reconstituted and denatured with 100 µL sodium deoxycholate (0.5%) in 50 mM ammonium bicarbonate. Heavy-labeled

APOL1 internal standard peptide was added (3 µL, 0.3 µM) and mixed at 25°C for 1 h at 1,000 rpm. Samples were reduced with 1 µL dithiothreitol (0.5 M), incubating for 30 min mixing at 1,000 rpm and 37°C in a Thermomixer. Cysteines were alkylated with 3 µL iodoacetamide (0.5 M) and incubated for 30 min in the dark. Proteins were digested with 5 µL trypsin (0.2 µg/µL) and incubated for 2 h at 37°C mixing at 1,000 rpm in an Eppendorf Thermomixer. The digestion was stopped with 4.4 µL HCl (5 M) and the samples were incubated at 4°C for 15 min to precipitate the sodium deoxycholate. After centrifugation at 15,000x*g* for 20 min at 4°C, the resulting supernatants were transferred to clean Eppendorf Lo-Bind tubes and stored at -20°C until analysis. Samples were thawed and the entire contents were transferred to a 96-well Oasis HLB prime µelution plate (Waters). The wells were washed 3 times with 100 µL 0.1% trifluouroacetic acid, then with 100 µL 0.5% formic acid. Peptides were eluted with 0.1% formic acid in 80% acetonitrile two times and dried-down using a CentriVap vacuum concentrator. The residues were reconstituted in 25 µL 0.1% formic acid/5% acetonitrile with mixing for 2 h at 1,400 rpm and room temperature. Samples were diluted with 25 µL 0.1% formic acid, vortexed, and centrifuged at 12,000x*g* and room temperature for 10 min, then 50 µL transferred to vials for injection. Finally, 40 µL of each sample was injected into the LC-MS/MS instrument. Concentrations of APOL1 protein in urine were estimated using the concentration of internal standard added to each sample.

Calibration and calculation of APOL1 protein concentrations

The LC-MS/MS assay of APOL1 in plasma was calibrated using external calibrators comprised of pooled human serum diluted into chicken serum, which provided a null- matrix similar to human and mouse serum samples.[4] The concentrations of APOL1 in the calibrators were estimated using standard addition of unlabeled peptides ALDNLAR and VTEPISAESGEQVER (4 µL of a 15 µM or 30 µM) at the end of digestion. The peak areas of the endogenous APOL1 peptides in the unspiked calibrators were divided by the peak areas of the spiked peptides and multiplied by the concentration of the spiked peptides. Results for the two spiked peptides were averaged. The concentration of each APOL1 protein isoform in each size fraction was estimated similarly, but instead used peak area ratios for the tryptic peptides LNILNNNYK for G0, LNMLNNNYK for G1, and LNILNNK for G2, multiplied by the known concentration of the corresponding spiked labeled peptide analogs, and then adjusted for ~20% trypsin digestion

efficacy for the inline digestion system (determined based on the digestion efficiency of the known concentration of intact apolipoprotein A-I and other proteins).

Transgenic mice

We generated Alb/APOL1 transgenic mice on the FVB/N background; these mice carry the human *APOL1* gene under the control of the murine albumin promoter, on the FVB/N background. These mice have an albumin-promoter driving a full length *APOL1* cDNA which contains His_6 tag sequence at 3'-terminus. We generated three strains: Alb/APOL1-G0, Alb/APOL1-G1 and Alb/APOL1 G2, each of which contains the coding sequences for one of these common *APOL1* genetic variants. The murine albumin enhancer/promoter region was cloned from mouse genomic DNA (C57BL/6J) by overlap extension PCR. The enhancer region includes sequence from chromosome 5 (90597809-90598158) and the promoter region includes sequence from chromosome 5 (bp 90608588-90608755). PCR products were flanked by Gateway recombinational cloning sites (attB4 on the left end, attB1r on the right end) and were recombined using Gateway BP cloning into an Entry vector using the manufacturer's protocols (ThermoFisher Scientific). The entire insert was sequence validated to confirm that no mutations were introduced. ApoL1 sequences were cloned with C-terminal His6 tags in standard Gateway attB1-attB2 Entry clones, and final transgenic constructs were generated by Gateway Multisite LR recombination of the albumin promoter Entry clone and the ApoL1 Entry clones into pDest-302, a Gateway Multisite vector containing attR4 and attR2 sites in a pUC19 backbone vector. Final clones were validated by restriction digest.

Mouse proteinuria model (triple intervention model)

All mouse experiments were conducted in accordance with the National Institutes of Health Guide for the Care and Use of Laboratory Animals and were approved in advance by the NIDDK Animal Care and Use Committee (Animal study proposal, K097-KDB-17 & K096-KDB-20). We studied both male and female mice, aged 8–10 weeks. Mice in experimental groups were matched for sex, age, and body weight. Random assignment to treatment groups were performed without regard to body weight. Sample sizes for the experiments were determined by referencing previous work with the same interventional model [5] without formal power calculations. Mice that died during the experimental period were excluded from analysis. We induced podocyte injury and proteinuria using a triple intervention approach, including administration of

interferon-γ, puromycin aminonucleoside, and basic fibroblast growth factor, as previously described [5]. Mice received injections on days -1 and +1 with interferon-γ (ProSpec, East Brunswick, NJ) at a dose of 10^6 U per kg body weight. On day 0, mice received puromycin aminonucleoside (Millipore-Sigma, Burlington, MA) by intraperitoneal administration, at a dose of 300 mg/kg body weight. On days 0 and 2, mice received basic fibroblast growth factor, 5 µg intravenously (Kaken Pharmaceutical, Tokyo, Japan), as previously described [5]. We measured glomerular filtration rate at day 10, as described below. We collected urine at day -2, day 7, and day 14 for 24 h using metabolic cages. On day 14, mice were euthanized by cervical dislocation after anesthesia with intraperitoneal 10% tribromethanol (Avertin) injection and after confirming loss of sensation by toe pinching and plasma, serum, kidneys, and liver were collected for analyte measurement and histologic analysis.

Mouse urinary biomarker measurements

We determined urine albumin levels with Albuwell M ELISA kits (Ethos Biosciences, Newtown Square, PA). Urine NGAL concentrations were measured using Mouse Lipocalin-2/NGAL DuoSet ELISA (R&D Systems, Minneapolis, MN). Urine creatinine concentrations were measured using the Creatinine Companion kit (Ethos Biosciences). Albuminuria and urinary NGAL were expressed as the ratio of each urine protein to urine creatinine concentrations. Investigators were not masked to group allocation but were masked when assessing outcomes.

*Measurement of mouse glomerular filtration rate (**GFR**)*

We measured GFR ten days after puromycin aminonucleoside injection. After anesthesia with 2.5% isoflurane, the dorsal hair was shaved and a fluorescence sensor and a battery pack (NIC-Kidney, Mannheim Pharma & Diagnostics, Mannheim, Germany) was placed on the dorsum and secured with surgical tape. FITC-sinistrin (Medi Beacon, St. Louis, MO) was dissolved in 100 µl PBS (15 mg/100g body weight) and injected intravenously or retro-orbitally. Fluorescent signals were measured for approximately one hour, then the sensor and battery were removed for analysis. Data were analyzed according to the manufacturer's instructions. Briefly, GFR was calculated using the half-life ($t_{1/2}$) derived from the rate constant of the single exponential elimination phase of the fluorescence-time curve and a semi-empirical mouse-specific conversion factor established previously [6].

$$\text{GFR}\ [\mu\text{L} / \text{min} / 100\text{g b.w.}] = \frac{14616.8\ [\mu\text{L} / 100\text{g b.w.}]}{\text{t}\ ½\ (\text{FITC} - \text{sinistrin})\ [\text{min}]}$$

In situ hybridization in mouse tissues

Chromogenic *in situ* detection of transgene RNA was performed on tissue microarrays prepared from formalin-fixed paraffin-embedded blocks, using RNAscope (Advanced Cell Diagnostics, Biotechne, Minneapolis, MN). Briefly, 5 µm tissue sections were de-paraffinized, boiled with RNAscope target retrieval reagent for 15 min and protease digested at 40°C for 30 min, followed by hybridization for 2 h at 40°C with RNA probe-Hs-*APOL1*-O1 (Catalog # 439871, Advanced Cell Diagnostics). RNA probe-Mm-PPIB (Catalog # 313911) and RNA Probe-DapB (Catalog # 310043) were used for positive and negative control, respectively. Specific probe binding sites were visualized using RNAscope 2.5 HD Reagent Kit [7] (Catalog # 322310). Fluorescent in situ detection of mRNA was also performed. RNA probe-Hs-APOL1-No-XMm (Catalog # 459791) and RNA probe- Mm-Nphs1 (Catalog # 433571) were used for hybridization. Specific probe binding sites were visualized using RNAscope Hiplex12 Reagents Kit (488, 550, 650) v2 (Advanced Cell Diagnostics) (Catalog # 324419).

Immunohistochemistry in mouse tissues

Mouse tissue was fixed with 10% buffered formalin for 24 h, embedded in paraffin and sectioned at 4-5 µm. The sections were deparaffinized/rehydrated, then antigen retrieval performed by heating in citrate-buffered medium for 15 min in hot water bath. Tissues were blocked by 2.5% normal horse serum. Sections were incubated for 1 h at room temperature with 5 µg/ml of primary antibody for APOL1 (5.17D12, rabbit monoclonal) that was kindly provided by Genentech (South San Francisco, CA) [8]. Sections were processed following ImmPRESS HRP Universal Antibody (Horse Anti-Mouse/Rabbit IgG) Polymer Detection Kit and ImmPACT DAB EqV Peroxidase (HRP) Substrate (Vector Laboratories, Burlingame, CA) protocol, and counter stained by hematoxylin.

Quantifying podocyte density in individual glomeruli

PodoCount, a computational tool for whole-slide podocyte quantification,[9] was used to detect, enumerate, and characterize podocyte nuclear profiles in the glomeruli of immunohistochemically labeled murine kidney sections. Formalin-fixed, paraffin embedded tissues (2 µm thickness) were immunohistochemically labeled for $p57^{kip2}$, a marker of podocyte

terminal differentiation (ab75974, Abcam, Cambridge, UK), and detected with horse radish peroxidase (RU-HRP1000, Diagnostic BioSystems, Pleasanton, CA) and diaminobenzidine chromogen substrate (BSB0018A, Bio SB, Santa Barbara, CA). A periodic acid-Schiff post-stain was applied without hematoxylin counterstain. The method uses a combination of stain deconvolution,[10] digital image processing,[11, 12] and simple feature engineering[13, 14] to compute histologic morphometrics[15, 16] from glomeruli and resident podocyte nuclei in whole-slide images of kidney specimens. PodoCount was used to assess four morphometrics in murine histology: podocyte nuclear count in each glomerulus, podocyte nuclear density, cumulative podocyte nuclear area, and podocyte nuclear coverage. PodoCount defines podocyte nuclear count as the number of podocyte nuclear profiles per glomerulus and cumulative podocyte nuclear area as the total area occupied by podocyte nuclei. These two metrics are further indexed to each glomerular cross-sectional area to provide estimates of glomerular podocyte spatial density.

Supplemental Table A. Transitions monitored for the quantification of total APOL1 in plasma.

Peptide	Haplotype Specificity	Transitions monitored and instrument settings (Plasma and urine concentrations)			Transitions monitored (Analysis of AF4 fractions)
		Transition	Cone (V)	Collision (kV)	
ALDNLAR	All	386.92/588.28	30	10	
ALDNLAR^ (heavy)		391.92/598.28	30	10	
VTEPISAESGEQVER		816.08/1091.64	44	32	815.9 / 1091.5
VTEPISAESGEQVER^ (heavy)		821.09/1101.58	44	32	820.9 / 1101.5
LNILNNK	G0	414.82/601.39	40	12	414.8 / 601.4
LNILNNK^ (heavy)		418.82/609.39	40	12	418.8 / 609.4
LNILNNNYK	G1	553.42/787.54	44	16	553.3 / 765.4
LNILNNNYK^ (heavy)		557.42/886.54	44	16	557.3 / 773.4
LNMLNNNYK	G2	562.45/896.56	36	15	562.3 / 765.4
LNMLNNNYK^ (heavy)		566.45/904.56	36	15	566.3 / 773.4

Heavy peptides each include a stable isotope labeled amino acid, either arginine (R^) or lysine (K^). The precursor and fragment mass-to-charge ratios monitored for each peptide (transitions: precursor/fragment) are listed for the assay run on plasma or urine and for the assay run on the fractions purified by asymmetric flow field-flow fractionation (AF4).

Supplemental Table B. Participant characteristics in the Seattle Kidney Study cohort.

	**SKS subcohort**		
**N, female**	160 (34.4%)		
**Age**	59 (49 - 67)		
**N, self-described Black race**	109 (23.4%)		
**N, self-described White race**	277 (59.6%)		
**N, self-described Other race**	79 (17.0%)		
**eGFR (mL/min per 1.73 m^2)**	41.6 (26.0 - 60.8)		
**ACR (mg/g)**	146.3 (16.8 – 750.4)		
**BMI (kg/m^2)**	30.4 (25.4 - 35.7)		
**Total Cholesterol (mg/dL)**	166 (138 - 202)		
**HDL Cholesterol (mg/dL)**	38.7 (31.3 – 48.8)		
**LDL Cholesterol (mg/dL)**	93.2 (76.1 – 121.8)		
**Triglycerides (mg/dL)**	118.0 (84.0 – 190.0)		
**N, current smokers**	109 (23.4%)		
**N, prevalent diabetes**	229 (49.2%)		
	**Black**	**White**	**Other**
**N, G0/G0**	42	276	74
**N, G0/G1**	31	0	2
**N, G0/G2**	14	1	3
**N, G1/G1**	10	0	0
**N, G1/G2**	7	0	0
**N, G2/G2**	5	0	0

A total of 465 participants from the Seattle Kidney Study with enough plasma for APOL1 measurement were included in this analysis. The numbers of people in each group are shown (N) along with the percent of the total in each group in parentheses. All other parameters are expressed as median (IQR). APOL1 genotypes were determined using mass spectrometry.

Supplemental Table C. Association of self-described race on APOL1 protein concentration in G0/G0 participants.

Race	Genotype	Plasma (µM)	HDL
**Black (n = 42)**	**G0/G0**	369.0 (307.9 - 490.0)	0.75 (0.44 - 1.09)
**White (n = 276)**	**G0/G0**	251.1 (201.2 - 314.7) ***	1.20 (0.74 - 1.67) ***
**Other (n = 74)**	**G0/G0**	269.4 (209.2 - 330.6) ***	1.33 (0.86 - 2.03) ***

Genotype was determined from the phenotype as identified by LC-MS/MS. Plasma concentrations of APOL1 were determined by LC-MS/MS and are expressed as median (interquartile range). The relative concentrations of APOL1 in the HDL fraction of plasma were determined previously [3] and are expressed as median (interquartile range). *** P-value <0.0001, Wilcoxon test, compared with Black participants.

Supplemental Table D. Associations between APOL1 concentration in urine with clinical variables.

	Univariate			Multivariate	
**Variable in Model**	**Beta**	**r**	**P-value**	**Beta**	**P-value**
**eGFR (CKD-EPI 2021) ***	-0.738	0.270	<0.001	-0.509	<0.001
**Albumin-to-creatinine ratio ***	0.345	0.224	<0.001	0.306	<0.001
**Age ***	-0.252	0.002	0.430	0.330	0.382
**Sex, Female**	0.046		0.798	-0.063	0.767
**BMI ***	-0.466	0.005	0.187	-0.665	0.078
**Current smoking**	-0.203		0.339	-0.225	0.285
**Prevalent diabetes**	0.203		0.235	0.164	0.381
**Cholesterol (mg/dL) ***	0.753	0.023	0.003	0.695	0.078
**HDL cholesterol (mg/dL) ***	0.409	0.009	0.069	-0.220	0.494
**Triglycerides (mg/dL) ***	0.172	0.004	0.194	-0.308	0.102
**Self-reported race ****					
**White**	-0.116		0.579	0.491	0.096
**Other**	0.371		0.184	0.470	0.173
**APOL1 genotype *****					
**G0/G1**	-0.041		0.903	-0.074	0.842
**G0/G2**	-0.087		0.849	-0.356	0.471
**G1/G1**	0.615		0.278	0.369	0.507
**G1/G2**	0.288		0.733	0.364	0.652
**G2/G2**	-0.141		0.884	0.370	0.736
**APOL1 in Plasma (nM) ***	0.505	0.012	0.030	0.817	0.006

Linear regression was used to assess the association of amount of APOL1 in urine (log-transformed) with clinical parameters. Clinical parameters that were log-transformed in the regression (P-value <0.05 by Shapiro-Wilk test) are denoted with an asterisk (*). Univariate regression included only one parameter in each model (race and genotype were modeled with dummy variables for multiple categories) and multivariate regression model included all of the parameters listed in the table. Statistically significant results (P<0.05) are highlighted in red. **Black race was used as the reference group for the regression of self-reported race. ***G0/G0 was used as the reference group for the regression of *APOL1* genotype.

Supplemental Figure A. Measured hydrodynamic diameter as function of fraction number. For each of the 40 fractions, the average hydrodynamic size of particles was determined by dynamic light scattering. Assay calibration was confirmed with certified particle size standards (20-100 nm). In some fractions with low particle numbers, hydrodynamic size was determined by interpolation.

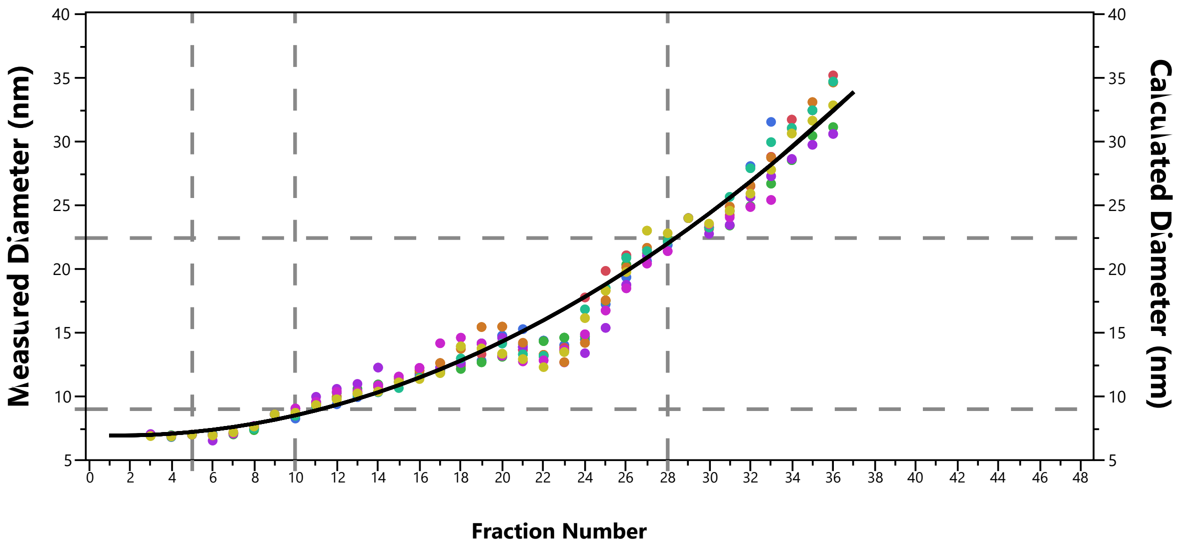

Supplemental Figure B. Lipoprotein particle size distribution in Black participants. Lipoprotein particles in plasma were separated using asymmetric flow field-flow fractionation and analyzed using LC-MS/MS. The relative amount of APOL1 protein in each fraction (%) is shown as mean ± SD. Bars are shaded different colors in order to more conveniently compare size fractions across genotypes.

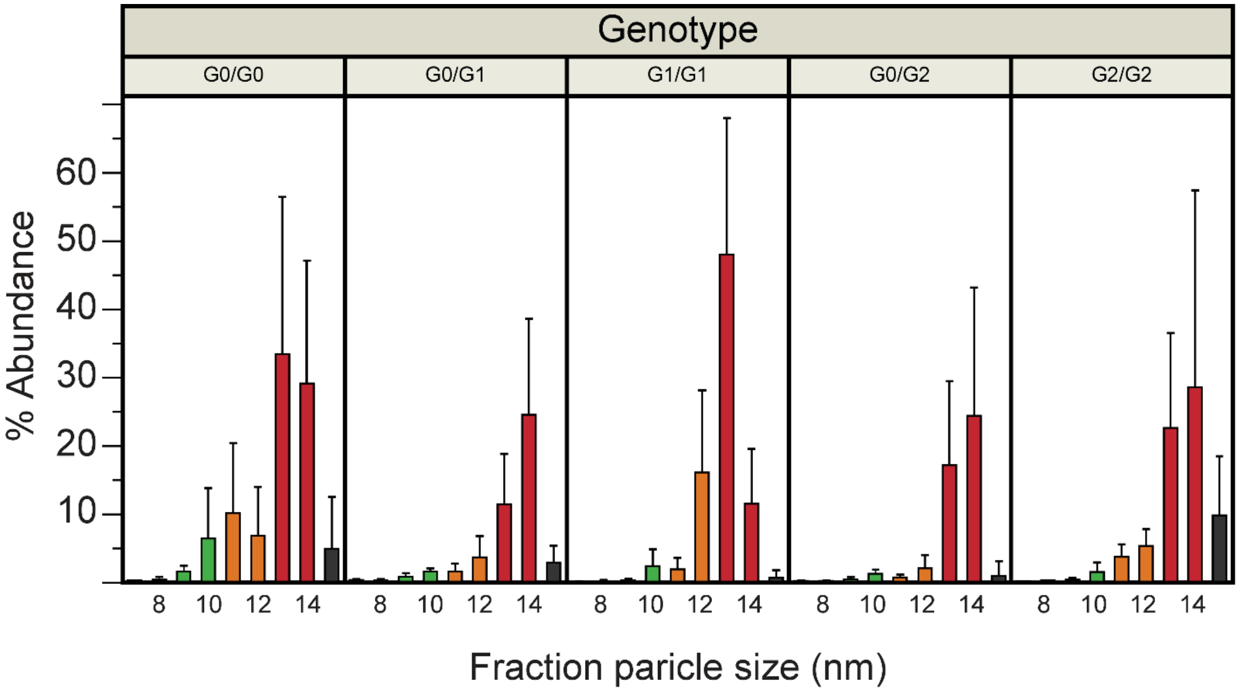

Supplemental Figure C. **Apolipoprotein size distribution determined by asymmetric flow field-flow fractionation.** Deconvolution of experimental size distribution profiles and calculated Gaussian components for apolipoproteins in representative samples separated by genotype. For APOL1, the profiles were generated from the sum of APOL1-G0, APOL1-G1 and APOL1-G2 peptides. APOL1-containing particles appear to be similar in size to ApoE-containing particles.

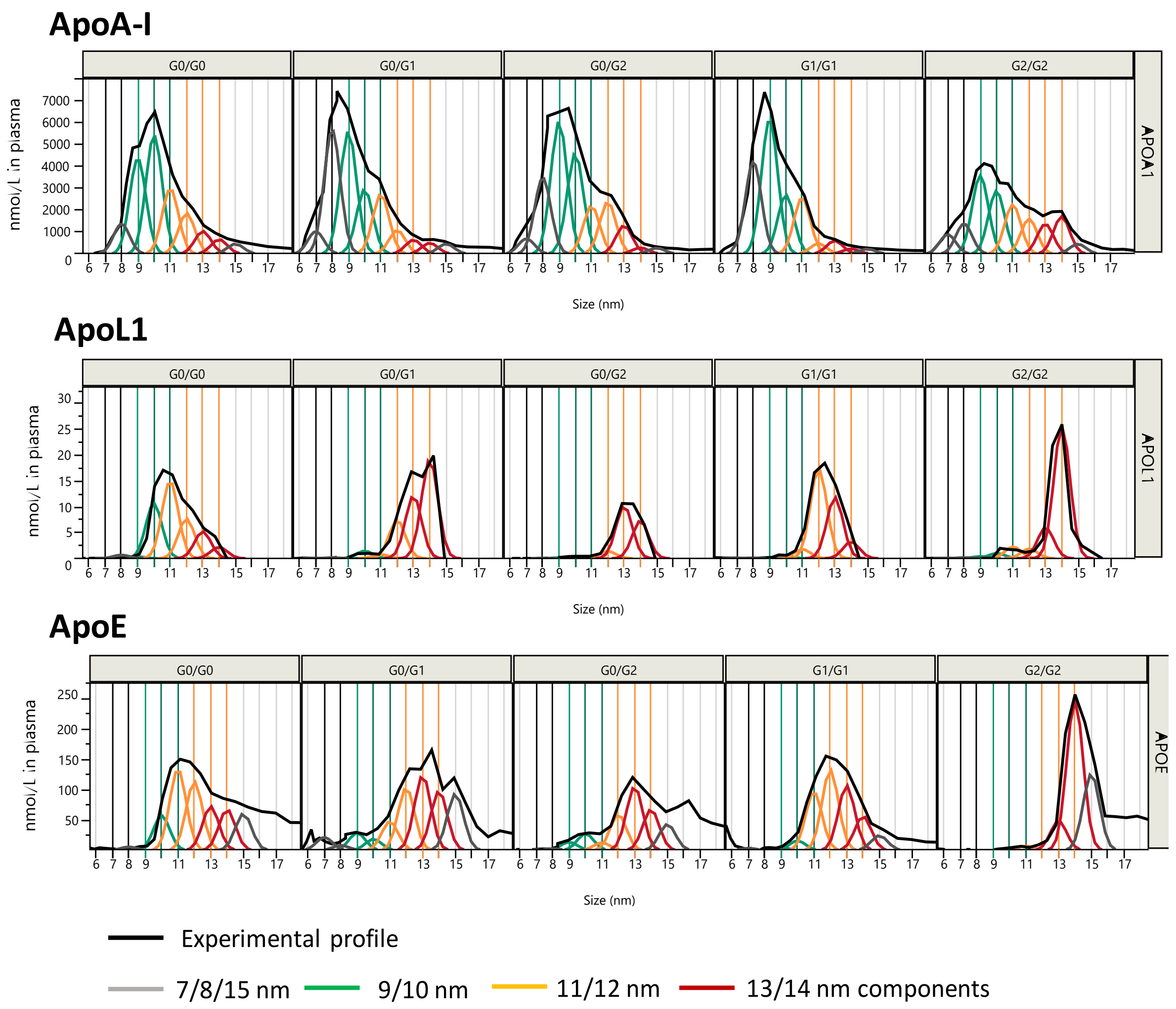

Supplemental Figure D. **Fluorescent in situ hybridization in liver, kidney, and spleen.** APOL1 (magenta) and Nphs1 (cyan) mRNA are detected along with a DAPI (blue) nuclear stain. APOL1 mRNA was detected in Alb/APOL1-G0, G1, G2 mouse liver, but not detected in kidney or spleen. Scale bars are 50 µm.

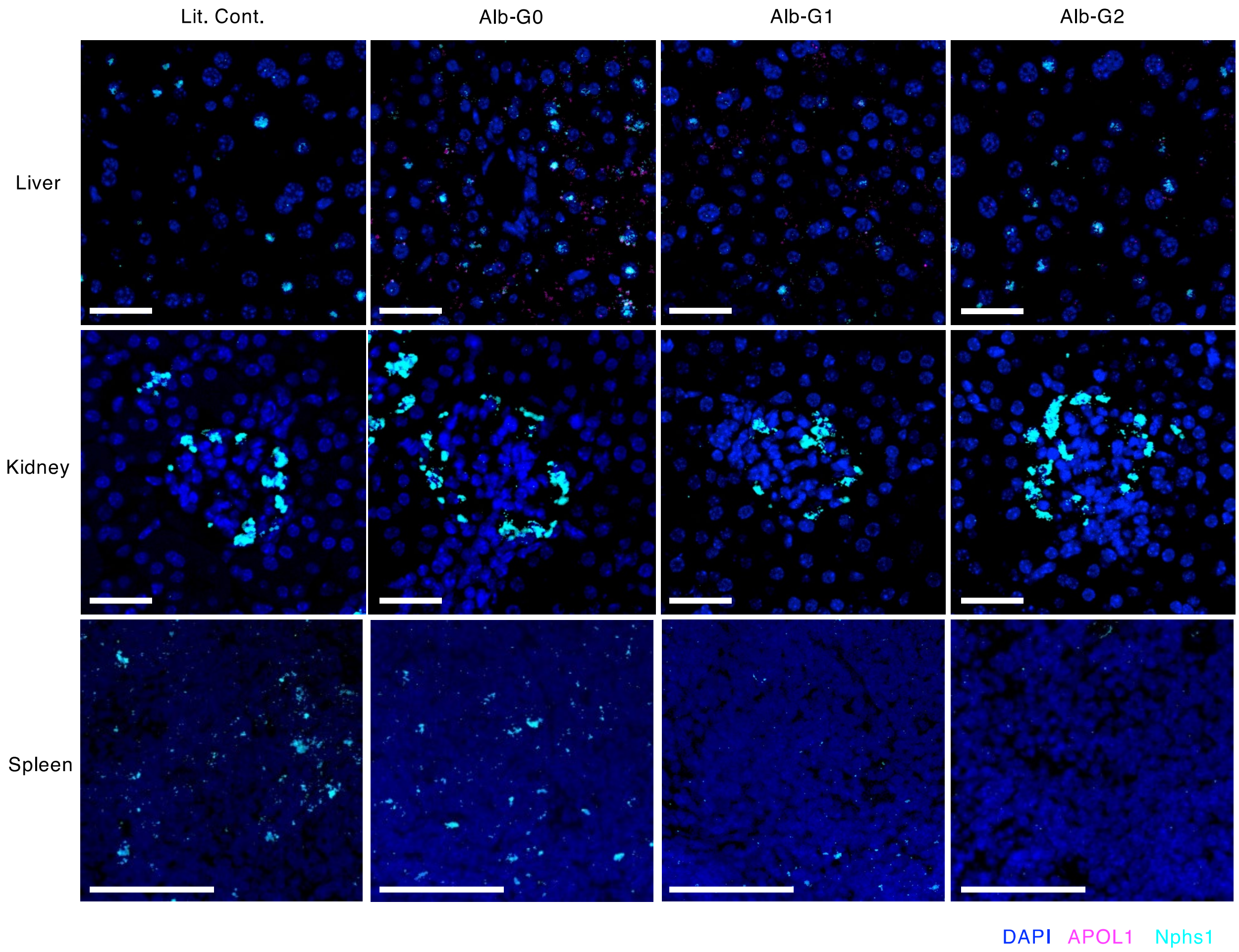

Supplemental Figure E. Concentration of APOL1 protein in mouse plasma before and after the triple intervention. Human APOL1 in mouse plasma was quantified using LC-MS/MS. Data are shown for mice that did not receive the triple intervention (left) as well as those that did (right; these are the same data as in Figure 2B, replotted to be on the same scale as mice that did not receive the triple intervention).

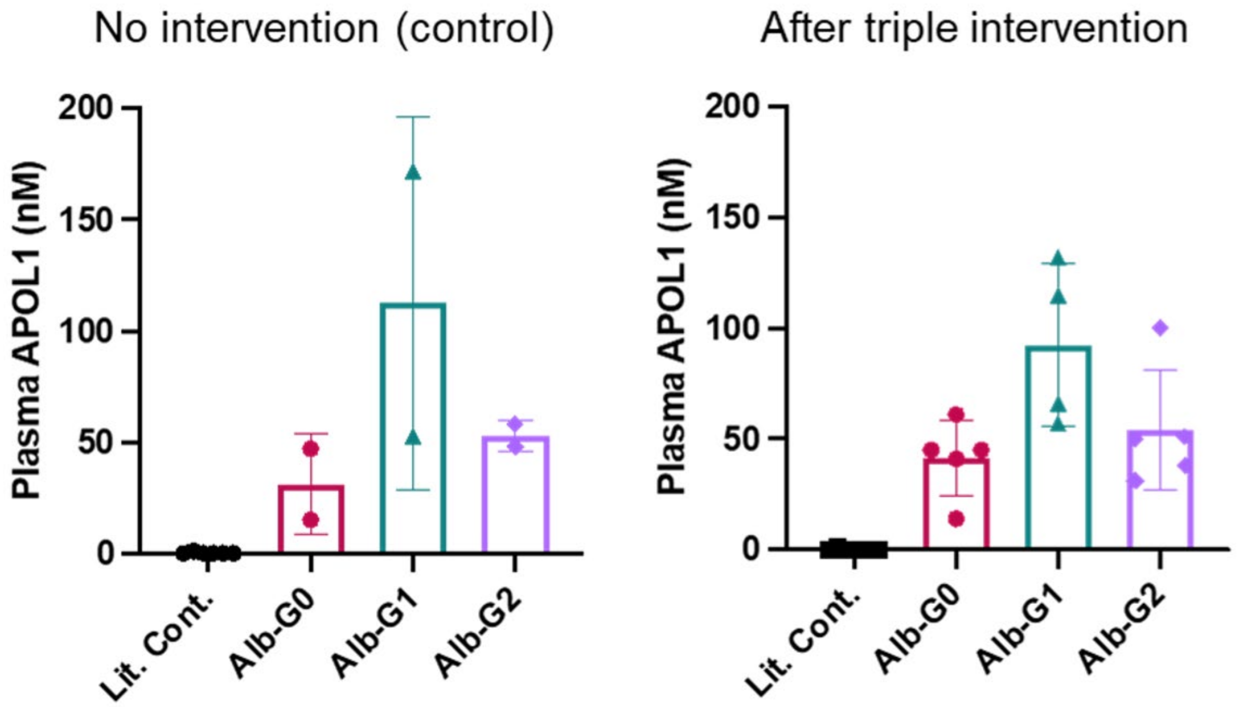

References for Supporting Information

1. Bosworth CR, Levin G, Robinson-Cohen C, Hoofnagle AN, Ruzinski J, Young B, et al. The serum 24,25-dihydroxyvitamin D concentration, a marker of vitamin D catabolism, is reduced in chronic kidney disease. Kidney Int. 2012;82(6):693-700. doi: 10.1038/ki.2012.193. PubMed PMID: 22648296; PubMed Central PMCID: PMC3434313.
2. Inker LA, Eneanya ND, Coresh J, Tighiouart H, Wang D, Sang Y, et al. New Creatinine- and Cystatin C-Based Equations to Estimate GFR without Race. N Engl J Med. 2021;385(19):1737-49. doi: 10.1056/NEJMoa2102953. PubMed PMID: 34554658; PubMed Central PMCID: PMC8822996.
3. Rubinow KB, Henderson CM, Robinson-Cohen C, Himmelfarb J, de Boer IH, Vaisar T, et al. Kidney function is associated with an altered protein composition of high-density lipoprotein. Kidney Int. 2017;92(6):1526-35. Epub 2017/07/30. doi: 10.1016/j.kint.2017.05.020. PubMed Central PMCID: PMCID: 5696089.
4. Henderson CM, Lutsey PL, Misialek JR, Laha TJ, Selvin E, Eckfeldt JH, et al. Measurement by a Novel LC-MS/MS Methodology Reveals Similar Serum Concentrations of Vitamin D-Binding Protein in Blacks and Whites. Clinical chemistry. 2016;62(1):179-87. Epub 2015/10/11. doi: 10.1373/clinchem.2015.244541. PubMed PMID: 26453697; PubMed Central PMCID: PMC4698095.
5. Okamoto K, Rausch JW, Wakashin H, Fu Y, Chung JY, Dummer PD, et al. APOL1 risk allele RNA contributes to renal toxicity by activating protein kinase R. Commun Biol. 2018;1:188. Epub 2018/11/13. doi: 10.1038/s42003-018-0188-2. PubMed PMID: 30417125; PubMed Central PMCID: PMC6220249.
6. Schreiber A, Shulhevich Y, Geraci S, Hesser J, Stsepankou D, Neudecker S, et al. Transcutaneous measurement of renal function in conscious mice. Am J Physiol Renal Physiol. 2012;303(5):F783-8. Epub 2012/06/15. doi: 10.1152/ajprenal.00279.2012. PubMed PMID: 22696603.
7. Aldigier JC, Kanjanbuch T, Ma LJ, Brown NJ, Fogo AB. Regression of existing glomerulosclerosis by inhibition of aldosterone. J Am Soc Nephrol. 2005;16(11):3306-14. Epub 2005/09/30. doi: 10.1681/ASN.2004090804. PubMed PMID: 16192423.
8. Scales SJ, Gupta N, De Maziere AM, Posthuma G, Chiu CP, Pierce AA, et al. Apolipoprotein L1-Specific Antibodies Detect Endogenous APOL1 inside the Endoplasmic Reticulum and on the Plasma Membrane of Podocytes. J Am Soc Nephrol. 2020;31(9):2044-64. doi: 10.1681/ASN.2019080829. PubMed PMID: 32764142; PubMed Central PMCID: PMC7461670.
9. Santo B, Rosenberg A, Sarder P. Automated Podocyte Quantification for Evaluation of Chronic Kidney Disease (1693). In “Abstracts from USCAP 2020: Medical Renal Pathology". Modern pathology : an official journal of the United States and Canadian Academy of Pathology, Inc. 2020;33(Suppl 2):1741-73. Epub 2020/03/07. doi: 10.1038/s41379-020-0479-2. PubMed PMID: 32139827; PubMed Central PMCID: PMC7104436.
10. Ruifrok AC, Johnston DA. Quantification of histochemical staining by color deconvolution. Anal Quant Cytol Histol. 2001;23(4):291-9. PubMed PMID: 11531144.
11. Gonzales R, Wintz P. Digital image processing. Reading, Mass.: Addison-Wesley Publishing Co, Inc.; 1987.
12. Veta M, van Diest PJ, Kornegoor R, Huisman A, Viergever MA, Pluim JP. Automatic nuclei segmentation in H&E stained breast cancer histopathology images. PLoS One. 2013;8(7):e70221. doi: 10.1371/journal.pone.0070221. PubMed PMID: 23922958; PubMed Central PMCID: PMC3726421.

13. Ginley B, Lutnick B, Jen KY, Fogo AB, Jain S, Rosenberg A, et al. Computational Segmentation and Classification of Diabetic Glomerulosclerosis. J Am Soc Nephrol. 2019;30(10):1953-67. doi: 10.1681/ASN.2018121259. PubMed PMID: 31488606; PubMed Central PMCID: PMC6779352.
14. van der Walt S, Schonberger JL, Nunez-Iglesias J, Boulogne F, Warner JD, Yager N, et al. scikit-image: image processing in Python. PeerJ. 2014;2:e453. doi: 10.7717/peerj.453. PubMed PMID: 25024921; PubMed Central PMCID: PMC4081273.
15. Kikuchi M, Wickman L, Hodgin JB, Wiggins RC. Podometrics as a Potential Clinical Tool for Glomerular Disease Management. Semin Nephrol. 2015;35(3):245-55. doi: 10.1016/j.semnephrol.2015.04.004. PubMed PMID: 26215862; PubMed Central PMCID: PMC4518207.
16. Venkatareddy M, Wang S, Yang Y, Patel S, Wickman L, Nishizono R, et al. Estimating podocyte number and density using a single histologic section. J Am Soc Nephrol. 2014;25(5):1118-29. doi: 10.1681/ASN.2013080859. PubMed PMID: 24357669; PubMed Central PMCID: PMC4005315.
